# Insights into *Streptomyces* spp. isolated from the rhizospheric soil of *Panax notoginseng*: isolation, antimicrobial activity and biosynthetic potential for polyketides and non-ribosomal peptides

**DOI:** 10.1186/s12866-020-01832-5

**Published:** 2020-06-03

**Authors:** Fei Peng, Meng-Yue Zhang, Shao-Yang Hou, Juan Chen, Ying-Ying Wu, Yi-Xuan Zhang

**Affiliations:** 1 School of Life Science and Biopharmaceutics, Shenyang Pharmaceutical University, Shenyang, 110016 People’s Republic of China; 2 Quanzhou Medical College, Quanzhou, People’s Republic of China

**Keywords:** *Streptomyces*, *Panax notoginseng*, Phylogenetic analysis, Type I polyketide synthases (PKS I), Type II polyketide synthase (PKS II), Nonribosomal peptide synthetases (NRPS)

## Abstract

**Background:**

Streptomycetes from the rhizospheric soils are a rich resource of novel secondary metabolites with various biological activities. However, there is still little information related to the isolation, antimicrobial activity and biosynthetic potential for polyketide and non-ribosomal peptide discovery associated with the rhizospheric streptomycetes of *Panax notoginseng*. Thus, the aims of the present study are to (i) identify culturable streptomycetes from the rhizospheric soil of *P. notoginseng* by 16S rRNA gene, (ii) evaluate the antimicrobial activities of isolates and analyze the biosynthetic gene encoding polyketide synthases (PKSs) and nonribosomal peptide synthetases (NRPSs) of isolates, (iii) detect the bioactive secondary metabolites from selected streptomycetes, (iv) study the influence of the selected isolate on the growth of *P. notoginseng* in the continuous cropping field. This study would provide a preliminary basis for the further discovery of the secondary metabolites from streptomycetes isolated from the rhizospheric soil of *P. notoginseng* and their further utilization for biocontrol of plants.

**Results:**

A total of 42 strains representing 42 species of the genus *Streptomyces* were isolated from 12 rhizospheric soil samples in the cultivation field of *P. notoginseng* and were analyzed by 16S rRNA gene sequencing. Overall, 40 crude cell extracts out of 42 under two culture conditions showed antibacterial and antifungal activities. Also, the presence of biosynthesis genes encoding type I and II polyketide synthase (PKS I and PKS II) and nonribosomal peptide synthetases (NRPSs) in 42 strains were established. Based on characteristic chemical profiles screening by High Performance Liquid Chromatography-Diode Array Detector (HPLC-DAD), the secondary metabolite profiles of strain SYP-A7257 were evaluated by High Performance Liquid Chromatography-High Resolution Mass Spectrometry (HPLC-HRMS). Finally, four compounds actinomycin X2 (**F1)**, fungichromin (**F2**), thailandin B (**F7**) and antifungalmycin (**F8**) were isolated from strain SYP-A7257 by using chromatography techniques, UV, HR-ESI-MS and NMR, and their antimicrobial activities against the test bacteria and fungus were also evaluated. In the farm experiments, *Streptomyces* sp. SYP-A7257 showed healthy growth promotion and survival rate improvement of *P. notoginseng* in the continuous cropping field.

**Conclusions:**

We demonstrated the *P. notoginseng* rhizospheric soil-derived *Streptomyces* spp. distribution and diversity with respect to their metabolic potential for polyketides and non-ribosomal peptides, as well as the presence of biosynthesis genes PKS I, PKS II and NRPSs. Our results showed that cultivatable *Streptomyces* isolates from the rhizospheric soils of *P. notoginseng* have the ability to produce bioactive secondary metabolites. The farm experiments suggested that the rhizospheric soil *Streptomyces* sp. SYP-A7257 may be a potential biological control agent for healthy growth promotion and survival rate improvement of *P. notoginseng* in the continuous cropping field.

## Background

The rhizosphere is a habitat where a high abundance of bacteria, actinobacteria and fungi, is observed. Those microbes are recruited by plant exudates released through the roots [1]. In this environment, they are exposed to a permanent state of competition; the production of specific antibiotics may play a decisive role in helping them survive within the rhizosphere microbial community [2]. Among them, it has been revealed that streptomycetes could help to promote the growth of host plants as well as to improve disease symptoms caused by plant pathogens through diverse mechanisms, including the production of bioactive metabolites, which are applied in the direct antagonism against pests and diseases [3, 4], in the changes of host physiological function [5], in the induction of host systemic acquired resistances [6], and so on. Among these characteristics, a significant common function for *Streptomyces* is the antibiotics production [7], which suggests that streptomycetes play a key role in the plant defense and are widely recognized for their potentials in the biocontrol [2]. For example, Blasticidin S, produced by *Streptomyces griseochromogenes*, was the first commercial antibiotic used in the control of rice blast in Japan [8]. *Streptomyces* sp. IA1, producing an antimicrobial actinomycin D, was isolated from Saharan soil in Amenas, Algeria. Moreover, strain IA1 and actinomycin D effectively reduced two fungal diseases, i.e., the Fusarium wilt of *flax* and chocolate spot of *field bean* [9]. *Streptomyces* sp. Acta 3034 from the rhizospheric soil of *Clitorea* sp., produced five new angucycline antibiotics, langkocyclines A1-A3, B1 and B2, which exhibited biological activities against Gram-positive bacteria and moderate inhibitory activities against several human tumor cell lines [10]. Streptomycetes may also promote the growth of the plant hosts by producing auxins that improve the root growth [11]. Therefore, members of the genus *Streptomyces* from rhizospheric soils are still a rich resource of secondary metabolites with various biological activities.

A diverse array of bioactive molecules are synthesized by the type I polyketide synthases (PKS I), type II polyketide synthase (PKS II or aromatic polyketide synthase) and nonribosomal peptide synthetases (NRPS) [12]. Degenerate PCR primers targeting on KS domain of PKS I and PKS II and A domain of NRPS have been applied into the screening of PKS and NRPS systems from sea sediments [13], cultured microorganisms including actinobacteria [14–16] and cyanobacteria [17]. To the best of our knowledge, data related to the functional genes responsible for secondary metabolites production in the soil-associated streptomycetes from the rhizosphere of *Panax notoginseng* are limited.

*Panax notoginseng* F. H. Chen (Araliaceae), named as Sanqi or Tianqi in China, is a well-known traditional Chinese medicine, which has wide application in promotion of blood circulation, removal of blood stasis, cure of cardiovascular diseases [18, 19]. The high-quality roots of *P. notoginseng* are obtained after growth for at least three years [20]. The long growth period easily results in the infection of soil-borne pathogens [21]. Up to now, several previous studies have been performed to investigate the rhizospheric soil and root endogenous microbial diversity during *P. notoginseng* cropping [22, 23]. In our long-term study, many bioactive bacteria and fungi with the production of many bioactive metabolites have been isolated from the environments related to *P. notoginseng* [24–27]. However, very little is known about the isolation, antimicrobial activity and biosynthetic potential for polyketide and non-ribosomal peptide discovery associated with the rhizospheric streptomycetes of *P. notoginseng*. Thus, the aims of the present study are to (i) identify culturable *Streptomyces* isolates from the rhizospheric soil of *P. notoginseng* by 16S rRNA gene, (ii) evaluate the antimicrobial activities and analyze the biosynthetic gene encoding polyketide synthases (PKSs) and nonribosomal peptide synthetases (NRPSs) of isolates, (iii) detect the bioactive secondary metabolites from the selected *Streptomyces* isolate, (iv) study the influence of the selected strain on the growth of *P. notoginseng* in the continuous cropping field. This study will provide a basis for the further discovery of the secondary metabolites from streptomycetes from the rhizospheric soil of *P. notoginseng* and their further utilization for biocontrol of plants.

## Results

### Diversity of streptomycetes from the rhizospheric soil of *P. notoginseng*

The method of Serial dilution spread plate was used to isolate actinobacteria from the rhizospheric soil of *P. notoginseng*. After 1–4 weeks, individual colonies were picked from the plates based on the morphological characteristics of aerial hyphae and substrate mycelia, into the fresh plates to obtain pure cultures. To further ascertain the taxonomic status of the isolates, the 16S rRNA gene was PCR-amplified and sequenced. Forty two representative isolates belonging to the genus of *Streptomyces* were selected based on 16S rRNA gene sequencing (Table S3). The percentages of 16S rRNA gene identity to the closest type strains were presented in Table S3. The 42 strains belonged to the genus of *Streptomyces* with the identities from 97.24 to 100% (Table S3). Among them, 34 isolates (81%) were from the rhizospheric soil of healthy *P. notoginseng*, whereas 4 isolates (9.5%) from the rhizospheric soil of root-rot *P. notoginseng* and 4 isolates (9.5%) from the rhizospheric soil with serious nematodes infection. The culturable *Streptomyces* strains showed more abundance in the healthy rhizospheric soil (81%) than in the sick soil (19%). On the other hand, the 16S rRNA gene sequences of strain SYP-A7053, SYP-A7096 and SYP-A8135 showed 97.24, 98.83 and 98.44% identity to *S. viridosporus* ATCC 27479 (DQ442556), *S. mexicanus* DSM 41796 (AB249966) and *S. caniferus* ATCC 43699 (AB184640), respectively. These strains are most likely to represent new species, and taxonomic studies are underway. These data indicated the considerable diversity of species grouped within the genus of *Streptomyces* isolated from the rhizospheric soil of *P. notoginseng*.

### Antimicrobial activity of the fermented extracts

All 42 streptomycetes were cultivated in two fermentation media, GYM4 and SPM1. 40 (95%) of 42 fermented extracts exhibited strong and moderate activities against at least one of the tested pathogens (Table 1). In addition, the extract of each strain from different fermentation broth exhibited obviously different activity. For example, only 29 (69%) of 42 extracts from GYM4 fermentation showed activities against at least one of the test pathogens, whereas 40 (95%) of 42 extracts from SPM1 fermentation displayed activities against at least one of the tested pathogens. Furthermore, most extracts from SPM1 displayed stronger activities than those from GYM4. In terms of the inhibition activity against *A. baumannii*, 16 strains (38%) in SPM1 exhibited inhibition activities, while only 1 strain (2.3%) in GYM4 had the same antagonistic activity. Moreover, whichever media was used, three isolates, SYP-A7053, SYP-A7193 and SYP-A7257, appeared to have a broad spectrum of antimicrobial activities. The strong inhibitory activities of these strains against the tested pathogens suggested that these soil-derived streptomycetes may be potential candidates for the production of bioactive metabolites under the optimal cultivation condition. These results also revealed that *Streptomyces* isolates and their metabolites with antagonistic activity may play an important biological protective role on the healthy growth of *P. notoginsen*.

**Table 1:** Antimicrobial activities and function genes of *Streptomyces* from the rhizospheric soil of *P. notoginseng*

Isolate no.	Activity against													Presence ^**c**^ of gene		
	***Staphylococcus aureus***^**a**^		***Escherichia coli***^**a**^		***Enterococcus faecium***^**a**^		***Proteus mirabilis***^**a**^		***Acinetobacter baumannii***^**a**^		***Achromobacter marplatensis***^**a**^		***Fusarium solani***^**b**^	PKS I	PKS II	NRPS
	GYM4	SPM1	GYM4	SPM1	GYM4	SPM1	GYM4	SPM1	GYM4	SPM1	GYM4	SPM1	SPM1			
**SYP-A7028**	**–**	**+++**	**+++**	**–**	**–**	**–**	**+++**	**–**	**–**	**–**	**–**	**++**	**–**	**+**	**+**	**–**
**SYP-A7030**	**+++**	**+++**	**–**	**–**	***+++***	***+++***	**–**	**–**	**–**	**–**	**–**	**+**	**+**	**+**	**+**	**–**
**SYP-A7031**	**–**	**–**	**–**	**+++**	**+**	**+++**	**–**	**+++**	**–**	**++**	**–**	**++**	**–**	**+**	**+**	**–**
**SYP-A7038**	**++**	**+++**	**–**	**+++**	**++**	**+++**	**–**	**+++**	**–**	**+**	**–**	**++**	**+**	**+**	**+**	**–**
**SYP-A7039**	**+++**	**+++**	***+++***	**–**	**–**	**+**	**–**	**++**	**–**	**–**	**–**	**++**	**+**	**+**	**+**	**–**
**SYP-A7049**	**++**	**+++**	**–**	**+++**	**++**	**+++**	**–**	**–**	**–**	**–**	**–**	**++**	**+**	**+**	**+**	**–**
**SYP-A7053**	**++**	**+++**	***+***	**+++**	**++**	**+++**	**++**	**+++**	**–**	**–**	**++**	**+++**	**+++**	**–**	**+**	**–**
**SYP-A7055**	**–**	**–**	**–**	**+**	**–**	**+++**	**–**	**–**	**–**	**+++**	**–**	**+**	**–**	**–**	**+**	**+**
**SYP-A7076**	**–**	***+++***	**–**	**–**	**–**	**–**	**–**	**–**	**–**	**–**	**–**	**+**	**++**	**+**	**+**	**+**
**SYP-A7077**	**–**	**–**	**–**	**+++**	**–**	**+++**	**–**	**+++**	**–**	**++**	**–**	**++**	**–**	**+**	**+**	**–**
**SYP-A7079**	**–**	**+**	**–**	**++**	**–**	**–**	**+**	**–**	**–**	**–**	**–**	**–**	**–**	**+**	**–**	**–**
**SYP-A7085**	**–**	***+++***	**+**	***++***	**–**	**–**	**–**	***+++***	**–**	**–**	**–**	**++**	**–**	**+**	**+**	**–**
**SYP-A7087**	***+++***	***+++***	**++**	**–**	**–**	**–**	***+***	**–**	**–**	**–**	**–**	**++**	**–**	**+**	**+**	**+**
**SYP-A7090**	**–**	**–**	**+**	***+***	**–**	**–**	**–**	**–**	**–**	**–**	**+++**	**++**	**+**	**–**	**+**	**–**
**SYP-A7096**	**–**	**–**	**–**	***+***	**–**	**–**	**–**	***+++***	**–**	**–**	**–**	**+++**	**+++**	**–**	**+**	**–**
**SYP-A7103**	***+++***	**–**	***+++***	***+++***	**–**	**–**	**–**	***++***	**–**	**–**	**–**	**+++**	**–**	**+**	**+**	**–**
**SYP-A7113**	**–**	***++***	**–**	***+***	**–**	**–**	**–**	***+***	**–**	**–**	**–**	**+++**	**–**	**–**	**+**	**–**
**SYP-A7114**	**–**	***++***	**–**	***+***	**–**	**–**	**–**	**–**	**–**	**–**	**–**	**++**	**–**	**–**	**+**	**–**
**SYP-A7131**	**–**	**–**	**–**	***+***	**–**	**–**	**–**	**–**	**–**	**–**	**–**	**+++**	**+**	**+**	**+**	**–**
**SYP-A7157**	**–**	***++***	**–**	**–**	**–**	**–**	**–**	**–**	**–**	**–**	**–**	**++**	**++**	**–**	**+**	**+**
**SYP-A7160**	**–**	**–**	**–**	***+++***	**–**	***+++***	**–**	**–**	**–**	**+++**	**–**	**+++**	**–**	**–**	**+**	**–**
**SYP-A7161**	**–**	**+**	**–**	**++**	**–**	**+++**	**–**	**–**	**–**	**+++**	**–**	**+**	**+++**	**+**	**–**	**–**
**SYP-A7178**	**++**	**+**	**–**	**++**	**–**	**++**	**–**	**–**	**–**	**++**	**–**	**++**	**++**	**+**	**+**	**–**
**SYP-A7185**	**+++**	**+**	**–**	**–**	**–**	**–**	**–**	**–**	**–**	**–**	**–**	**++**	**+**	**+**	**–**	**–**
**SYP-A7193**	**++**	**+++**	**+++**	**+++**	**+++**	***+++***	**–**	***+++***	**–**	**+++**	**–**	**+++**	**–**	**–**	**+**	**+**
**SYP-A7200**	**+++**	**–**	**–**	**–**	**+++**	**–**	**–**	**–**	**–**	**–**	**–**	**++**	**–**	**+**	**+**	**–**
**SYP-A7201**	**–**	**–**	***+++***	**–**	**–**	**–**	**+**	**–**	**–**	**–**	**–**	**–**	**–**	**+**	**+**	**–**
**SYP-A7212**	***++***	**–**	**+++**	**+++**	**–**	**–**	**–**	***+++***	**–**	**–**	**–**	**+++**	**+**	**–**	**+**	**–**
**SYP-A7234**	**+++**	***++***	**–**	***+***	***++***	***+++***	**–**	**–**	**+++**	**+++**	**–**	**+++**	**–**	**+**	**+**	**–**
**SYP-A7255**	***++***	***++***	**+++**	***++***	***+***	***+***	**–**	**–**	**–**	**+**	**–**	**–**	**–**	**+**	**+**	**+**
**SYP-A7257**	***++***	***++***	**–**	***+++***	***++***	***+++***	**–**	**–**	**–**	**+++**	**–**	**++**	**–**	**+**	**+**	**–**
**SYP-A7260**	**–**	**+++**	**–**	***+++***	**–**	**–**	**–**	**–**	**–**	**–**	**–**	**+++**	**–**	**+**	**+**	**–**
**SYP-A7261**	**–**	**++**	**–**	**–**	***+***	***+++***	**–**	**–**	**–**	**+++**	**–**	**–**	**–**	**+**	**+**	**–**
**SYP-A7283**	***++***	***++***	**–**	**–**	**–**	***+++***	**–**	**–**	**–**	**+++**	**–**	**–**	**+++**	**+**	**+**	**–**
**SYP-A7284**	**+++**	**–**	**–**	**–**	***+++***	**–**	**–**	**–**	**–**	**–**	**–**	**–**	**–**	**+**	**+**	**–**
**SYP-A7748**	**–**	**–**	**–**	**–**	**–**	**–**	**–**	**–**	**–**	**–**	**–**	**–**	**–**	**–**	**+**	**–**
**SYP-A7750**	**++**	**+**	**–**	**–**	***+++***	***+*+**	**–**	**–**	**–**	**++**	**–**	**++**	**+**	**+**	**+**	**+**
**SYP-A7752**	**–**	**–**	**–**	**–**	**–**	**–**	**–**	**–**	**–**	**–**	**–**	**–**	**–**	**–**	**–**	**–**
**SYP-A8135**	***+***	**–**	**–**	**–**	***+++***	**–**	**–**	**–**	**–**	**–**	**–**	**–**	**–**	**+**	**+**	**–**
**SYP-A8136**	***+***	***++***	**–**	**–**	**–**	***+++***	**–**	**–**	**–**	**+++**	**–**	**+**	**–**	**+**	**+**	**–**
**SYP-A8194**	**+++**	**+++**	**–**	**–**	***+++***	***+++***	**–**	**–**	**–**	**+++**	**–**	**+**	**–**	**+**	**+**	**–**
**SYP-A8195**	**–**	***+++***	**–**	**–**	**–**	**+**	**–**	**–**	**–**	**–**	**–**	**++**	**–**	**+**	**+**	**–**

^a^ Ratings: -, % inhibition ≤50; +, % inhibition ≥50; ++, % inhibition ≥70; +++, % inhibition ≥90

^b^ Estimated by measuring the diameter of the clear zone of growth inhibition. Symbols: -, no activity; +, between 4 and 6 mm; ++, between 7 and 9 mm and +++, between 10 and 12 mm; weak activity, moderate activity, and strong activity, respectively

^c^ +, present; −, absent

### Distribution and phylogenetic analysis of biosynthetic genes

All 42 *Streptomyces* isolates were screened for the presence of KS domain related to PKS I and PKS II genes and A domain associated with NRPS genes. In total, 41 (98%) of 42 strains yielded sequence-verified genes related to at least one of three targeted genes (Table 1). More specifically, PKS I loci were detected in 30 strains (71%), PKS II loci in 38 strains (90%) and NRPS loci in 7 strains (17%). Meanwhile, 30 strains (71%) had at least two types of three genes, and 4 strains (9%), SYP-A7076, SYP-A7087, SYP-A7255 and SYP-A7750 possessed three types of the biosynthetic genes simultaneously (Table 1). These results suggested that the biosynthetic genes of PKS I and PKS II were widespread in the rhizospheric soil-derived streptomycetes of *P. notoginseng*.

**Fig. 1:**
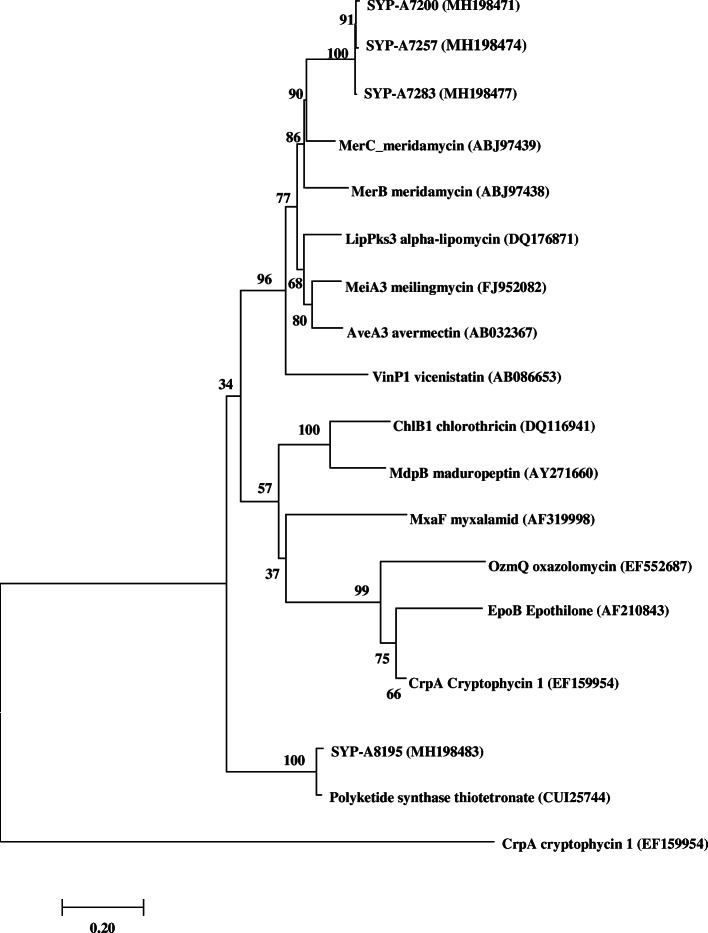
Neighbor-joining tree of KS amino acid sequences of isolates from this study and reference sequences of known representative PKS I. The scale bar indicates 0.20 substitutions that occur per site. Percentage bootstrap values of neighbor-joining analysis from 1000 resamplings are indicated at the nodes

Using BlastX search of GenBank, the PKS I sequence shared 93–100% sequence similarity with their closest matches (Table S4). Among them, KS sequences of 20 strains showed the high similarities at the amino acid level with that of *Bacillus* sp. from 98 to 100% (Table S4). PKS-I sequences retrieved from SYP-A7200, SYP-A7257 and SYP-A7283 clustered with a gene product (MerC) involved in the production of meridamycin [28] with the bootstrap value of 90% (Fig. 1). In addition, PKS-I sequences from *Streptomyces* strains SYP-A8195 showed the highest identity (96%) to the PKS-I gene responsible for biosynthesizing thiotetronate antibiotics [29], forming a separate clade with the bootstrap value of 100% (Fig. 1, Table S4).

**Fig. 2:**
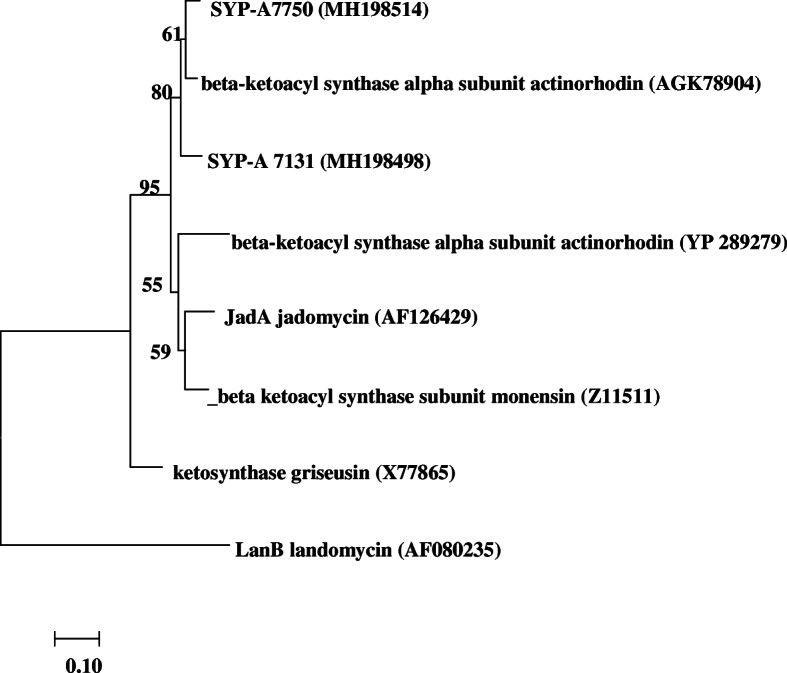
Neighbor-joining tree of KS amino acid sequences of isolates from this study and reference sequences of known representative PKS II. The scale bar indicates 0.10 substitutions that occur per site. Percentage bootstrap values of neighbor-joining analysis from 1000 resamplings are indicated at the nodes

The vast majority (38 isolates, 90%) of the KS sequences are most closely related to modular PKS II genes according to amino acid identities and phylogenetic analyses (Fig. 2, Table S4). All of the 38 KS sequences had the amino acid identities ranging from 77 to 100% (Table S4). Among them, SYP-A7131 and SYP-A7750 shared 90 and 94% amino acid identities (Table S4) with the sequence involved in the production of actinorhodin [30], respectively, and also formed the separated clade supported by 80% of bootstrap values. The A domains related to NRPS were sequenced for analyzing their phylogenetic relationships (Table S4). Only 7 sequences had the correspondence BLAST matches to the A domain with the amino acid identities ranging from 92 to 97%.

### Secondary metabolite profiles of fermentation extracts of the isolates [44]

**Fig. 3:**
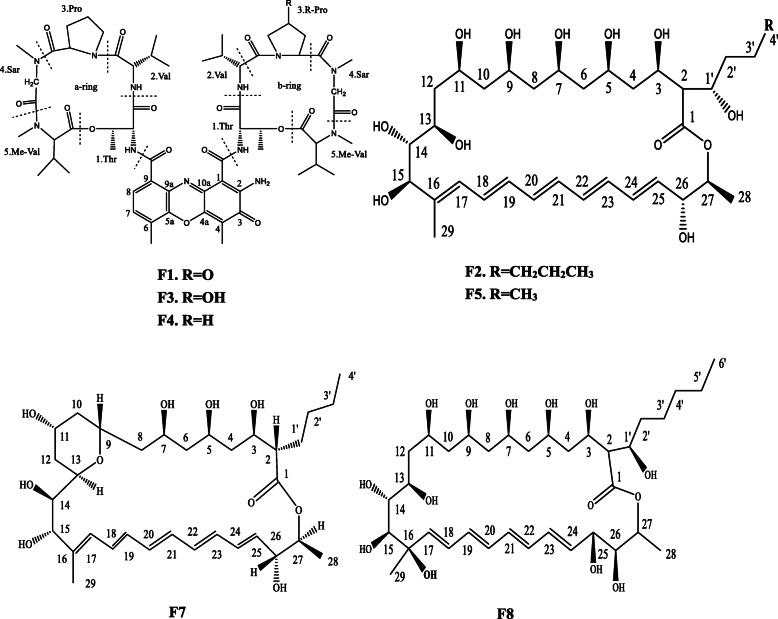
Chemical structures of secondary metabolites of strain SYP-A7257

Based on the functional gene screening and antimicrobial activities, 8 strains were selected for small-scale fermentation and HPLC-DAD analysis, among which 6 strains (SYP-A7200, SYP-A7257, SYP-A7283, SYP-A7131, SYP-A7750, SYP-A8195) (marked with * in Table S3) grouped with the described functional chemical classes in phylogenetic relationship analysis and 2 strain, SYP-A7053 and SYP-A7193, had a broad spectrum of antimicrobial activities. Under investigating the spectra of culture extracts by HPLC–DAD, strain SYP-A7257 exhibited a characteristic chemical profile differing from another 5 strains with characteristic UV/Vis absorption at bands of 325 to 360 nm and 241 to 446 nm, indicating the chromophore of typical polyene macrolide and of typical actinomycin analogues, respectively (Figure S1). Therefore, the extract of strain SYP-A7257 was analyzed by HPLC-HRMS to predict the numbers and diversity of compounds with respect to the presence of PKS and NRPS biosynthetic genes. HPLC-HRMS analysis of the extract of strain SYP-A7257 (Figure S2, S3-S8) showed the total ion current (TIC) chromatogram with six main peaks in the positive mode at m/z 1269.6146 (**F1**), 693.3818 (**F2**), 1293.6149 (**F3**), 1277.6161 (**F4**), 665.3505 (**F5**) and 709.3771 (**F6**), respectively (Table 2). According to the UV/Vis, MS and restricting to known compounds produced by *Streptomyces* spp., these compounds could be preliminarily identified as follows: **F1** as actinomycin X_2_, **F2** as fungichromin, **F3** as actinomycin X_0β_, **F4** as actinomycin D, **F5** as 1′,14-dihydroxyisochainin, **F6** as hydrofungichromin, respectively (Fig. 3 and Table 2). Among them, compound **F6** represents yet unknown derivative of fungichromin with [M + Na]^+^ at m/z 709.3771 (C_35_H_58_NaO_13_), indicating that compound **F6** possessed an additional hydroxyl group compared to fungichromin (C_35_H_58_NaO_12_, [M + Na]^+^ at m/z 693.3818). This compound did not match with any published compounds in spite of having the same UV spectra with that of fungichromin (Figure S8). Thus, strain SYP-A7257 was selected as the target strain for further chemical and bioactive characterization.

**Table 2:** The secondary metabolites from the selected isolate SYP-A7257 analyzed by LC-MS

No.	Retention time (min)	Molecular formula	Ion molecular formula	m/z	m/z (Calc)	Deviation (ppm)	Compound	UV (CH_**3**_CN-H_**2**_O) (nm)	Structural class	Reference (s)	Activity
**F5**	**25.688**	**C**_**33**_**H**_**53**_**O**_**12**_	**C**_**33**_**H**_**54**_**NaO**_**12**_	**665.3505**	**665.3507**	**0.37**	**1′, 14-Dihydroxyisochainin**	**325, 340, 360**	**polyene macrolides**	[31]	**antifungal**
**F6**	**28.963**	**C**_**35**_**H**_**57**_**O**_**13**_	**C**_**35**_**H**_**58**_**NaO**_**13**_	**709.3771**	**709.377**	**−0.19**	**hydrofungichromin**	**325, 340, 360**	**polyene macrolides**	**New compound**	**ND**
**F2**	**31.555**	**C**_**35**_**H**_**57**_**O**_**12**_	**C**_**35**_**H**_**58**_**NaO**_**12**_	**693.3818**	**693.382**	**0.36**	**fungichromin**	**325, 340, 360**	**polyene macrolides**	[32]	**antifungal**
**F3**	**40.872**	**C**_**62**_**H**_**85**_**N**_**12**_**O**_**17**_	**C**_**62**_**H**_**86**_**N**_**12**_**NaO**_**17**_	**1293.6149**	**1293.6126**	**−1.77**	**Actinomycin X**_**0β**_	**241, 446**	**PKS-NRPS hybrid**	[33]	**antibacteria**
**F1**	**42.672**	**C**_**62**_**H**_**84**_**N**_**12**_**O**_**17**_	**C**_**62**_**H**_**85**_**N**_**12**_**O**_**17**_	**1269.6146**	**1269.615**	**0.33**	**Actinomycin X2**	**241, 446**	**PKS-NRPS hybrid**	[33]	**antibacteria**
**F4**	**43.398**	**C**_**62**_**H**_**85**_**N**_**12**_**O**_**16**_	**C**_**62**_**H**_**86**_**N**_**12**_**NaO**_**16**_	**1277.6161**	**1277.6177**	**−4.39**	**Actinomycin D**	**241, 446**	**PKS-NRPS hybrid**	[33]	**antibacteria**

*ND*, not determined because of insufficient amounts of compounds

### Identification of secondary metabolites and their antimicrobial activities

To further confirm chemical structures of metabolic products, a serial of separation and purification were performed in the crude extract of strain SYP-A7257, and two compounds, corresponding to the HPLC-HRMS analysis, were obtained. Unfortunately, compound **F6** was not obtained because of its low production in the extract. Compound **F1** (Fig. 3) was isolated as a dark red amorphous powder with molecular weight of 1269.0 displaying maximal absorbance at 241 nm (shoulder) and 446 nm similar to those of known actinomycin derivates (Fig. S1). ESI-MS of this compound revealed molecular ion peak at m/z 1292.0 [M + Na]^+^ (Fig. S9), which was identical to that of actinomycin X_2_ further confirmed on the basis of ^1^H NMR and ^13^C NMR (Figure S10 and S11; Table S5) [33]. The other compound **F2** (Fig. 3) exhibited a typical characteristic of polyene macrolides spectra (λmax 360, 330 and 325 nm) (Fig. S1). ESI-MS analysis suggested its molecular masses to be m/z 669.4 [M-H]^−^ (Fig. S12) and revealed its molecular formula to be C_35_H_58_O_12_, according to the ^1^H and ^13^C NMR spectral data (Figure S13 and S14; Table S6). Thus, compound **F2** was identified as a known polyene antibiotics fungichromin [32].

To attain more antimicrobial compounds from strain SYP-A7257, a larger scale (10 L) extraction from culture grown in SPM1 medium was performed and 30.0 g of the crude material was attained. Compounds **F7** and **F8** were obtained under a series of separation and purification procedures. The structures of **F7** and **F8** (Fig. 3) were determined by using data from MS, and NMR techniques (Table S7). Compound **F7** was obtained as a pale yellowish powder. The molecular mass of **F7** was determined by ESI-MS, which gave the mass of m/z 607.4 [M-H]^−^ (Fig. S15) and revealed its molecular formula to be C_33_H_52_O_10_ in accord with the ^1^H and ^13^C NMR spectral data (Fig. S16 and S17; Table S7). Thus, compound **F7** was identified as a known polyene macrolide thailandin B [34]. Compound **F8** was identified as a polyene macrolide by a diode array detector (DAD)/UV spectra with three characteristic λmax at 289, 303, 319 nm. Positive ESI-MS of **F8** showed the molecular ion [M + H]^+^ at m/z 705.5 (Fig. S18) and revealed its molecular formula C_35_H_60_O_14_ in accord with the ^1^H and ^13^C NMR spectral data (Figure S19 and S20; Table S7). Thus, compound **F8** was identified as a known polyene macrolide antifungalmycin [35].

The results from the antimicrobial assays showed that compound **F1** displayed strong activity against *S. aureus* with MIC value of 0.3 μg/ml and weak activities against other test strains (Table 3). Compounds **F2**, **F7** and **F8** exhibited weak activities against all of the test bacteria. However, they had strong activities against pathogenic fungi *C. albicans* with MIC values of 1.24, 1.25 and 1.10 μg/ml, respectively (Table 3).

**Table 3:** MICs of compound F1, F2, F7 and F8 against pathogenic bacteria and fungi

MICs (μg/ml)							
Compound	***Staphylococcus sureusa***	***Escherichia coli***	***Enterococcus faecium***	***Proteus mirabilis***	***Acinetobacter baumannii***	***Achromobacter marplatensis***	***Candida albicans***
**F1**	**0.3**	> 200	27.2	> 200	134.5	14.6	21.6
**F2**	34.0	148.1	184.8	21.5	34.3	49.4	**1.24**
**F7**	> 200	> 200	> 200	> 200	> 200	> 200	**1.25**
**F8**	> 200	> 200	> 200	> 200	> 200	> 200	**1.10**
**Vancomycin**	2.5	50.0	10.0	50.0	30.0	50.0	–
**Geneticin**	–	–	–	–	–	–	6.0

### The farm experiment results

The seedling survival and healthy growth of *P. notoginseng* in the continuous cropping field are very important for *P. notoginseng* planting. The farm experiment showed that the plants in SYP-A7257 treatment (T) group grew healthily with high stems and wide leaves of dark green, while plants in control (CK) group grew slowly and were lack of vigor. The dead seedlings observed usually presented as root-rot diseases. The average survival rates in T groups at every recorded time (30, 60, 90 and 120 days) were significantly higher than those in CK groups (*P* < 0.05). On 120 days after the first-spray, the average emergence rate in T plots was 83.0%, while 68.0% in CK plots (Table 4), indicating that strain SYP-A7257 may have protective effects on the healthy growth of *P. notoginseng* against root-rot disease in the continuous cropping field.

**Table 4:** The average seedling survived rate of *Panax notoginseng* with the treatment of strain SYP-A7257 in continuous cropping field

Treatment	Average survival rate of ***Panax notoginseng***				
	0 day	30 days	60 days	90 days	120 days
**Control**	91 ± 2	76 ± 2	69 ± 2	68 ± 2	68 ± 1
**SYP-A7257**	93 ± 2	89 ± 1	84 ± 2	83 ± 3	83 ± 1

**Note:** The values are the mean of three biological replicates, 3 plots of 400 seedlings for both control and strain SYP-A7257 sprayed plants. The numbers are the percentage of seedling survivors with a statistically significant difference according to the one-way analysis of variance (*P* < 0.05)

## Discussion

Although members of *Streptomyces* have long been extensively screened for bioactive compounds, the emergence of drug-resistant bacteria and the relative ease of generating genome sequences have revived the importance of streptomycetes as producers of new natural products with various biological activities and resulted in renewed efforts in isolating these strains from the untapped environments [36]. Streptomycetes are Gram-positive bacteria ubiquitously living in soil, where they are the largest genus of *Streptomycetaceae* family (order *Actinomycetales*), consisting of nearly 600 formally described species [37], http://www.ezbiocloud.net). Many species are not pathogenic to human or the plants [36]. On the contrary, streptomycetes have ability to produce many enzymes and bioactive secondary metabolites [7]. All these substances give them potential for application in plant growth promotion and the biocontrol [36]. Thus, it has been assumed that high levels of antagonistic streptomycetes derived from naturally-occurring soils have a significant effect on the disease suppression [38]. Thus, antagonistic streptomycetes derived from rhizospheric soil are still an important source of novel natural products for chemical defense of plants. *P. notoginseng* as perennial plants is mainly cultivated for several years, which leads to infection by soil-derived pathogens including fungi, bacteria and nematodes [21]. There are many studies about exploring diversity of microorganisms from different environments and investigating the antagonistic activity of bacteria isolated from those environments, such as *Bacillus* spp. used as biocontrol agents [21–23]. However, the comprehensive investigation has been rarely performed on the isolation, antimicrobial activity and biosynthetic potential for polyketides and non-ribosomal peptide discovery associated to the *Streptomyces* strains, residing in the rhizospheric soil of *Panax notoginseng*.

In the present study, we have obtained 42 streptomycetes strains from 12 rhizospheric soil samples of *P. notoginseng*, and then these strains were subjected to the 16S rRNA gene analysis to ascertain their taxonomic relationships (Table S3). The culturable streptomycetes strains showed more abundance in the rhizospheric soil of healthy *P. notoginseng* (81%) than that of sick *P. notoginseng* (19%), which indirectly implied that the most species of the genus *Streptomyces* are beneficial to plant health [39]. To our knowledge, this is the first research of culturable *Streptomyces* strains from soil environments of *P. notoginseng* in Wenshan region of Yunnan province in China.

It is important to explore these streptomycetes having antibacterial and antifungal activities as producers of novel natural products or for direct use as biocontrol in soil environments of *P. notoginseng* [36]. Therefore, these isolates were evaluated for their antagonistic activities under two culture conditions. The antimicrobial experiments showed most of the *Streptomyces* strains gave high percentage of inhibition to all the tested pathogens. For example, isolate SYP-A7053 identified as *Streptomyces viridosporus* showed significant antifungal activity against *F. solani* (11.8 mm). Similarly, isolate SYP-A7193 isolated from the rhizospheric soil of the healthy *P. notoginseng*, showed significant antimicrobial activity against *S. aureus*, *E. coli*, *E. faecium*, *P. mirabilis*, *A. baumannii* and *A. marplatensis*. Interestingly, when employing medium SPM1 as fermentation broth, these isolates displayed higher inhibition activity against all the tested pathogens than those cultivated in medium GMY4. Similar finding was reported by Wu et al. (2018) [40], who used a cultivation-dependent procedure to change media type, resulting in the discovery of inthomycin B from a marine-derived *Streptomyces* sp. YB104. A recent study also revealed that a near-shore marine intertidal zone-associated *Streptomyces* strain (USC-633) was cultivated under a series of complex media to determine optimal parameters leading to the secretion of various array of secondary metabolites with antimicrobial activities against diversity drug-resistant bacteria [41]. Thus, our study indicates that these streptomycetes have potential ability to synthesize bioactive metabolites as defensive substances under different culture conditions. Their antagonistic activities to the plant pathogenic bacteria (*A. marplatensis*) and fungi (*F. solani*) also play a solid foundation for these soil-derived *Streptomyces* strains as biocontrol agents for *P. notoginseng* cultivation in the future. Thus, the selected strain SYP-A7257, which produced the antibiotics actinomycin and polyene macrolides derivates with the antibacterial and antifungal activity, respectively, was applied to study the biocontrol for *P. notoginseng* growth in the continuous cropping field. To the best of our knowledge, continuous cropping obstacles significantly affect *P. notoginseng* survival and cause severe mortality to plants [42]. In our farm experiments with continuous cropping soil, *Streptomyces* SYP-A7257 had showed desirable biocontrol potential for *P. notoginseng* healthy growth and improvement of the survival rates compared to CK plots without strain spray (Table 4), which suggested that *Streptomyces* sp. SYP-A7257 may be a promising biocontrol agent against root-rot disease to promote plants healthy growth because of the chemical defense substances secreted by this strain.

To the best of our knowledge, the majority of compounds from actinobacteria are demonstrated to be complex polyketides and non-ribosomal peptides [43]. It may be hypothesized that a genome with a vast number of biosynthetic gene clusters is more likely to lead to a positive hit under an PKS/NRPS gene screening procedure [43]. In our study, PCR screening of 42 soil-derived streptomycetes revealed that genes associated with PKS I and PKS II biosynthesis were widespread and generally distributed among different species of the genus *Streptomyces*, which was consistent with the viewpoint of Schneemann et al. (2010) [43]. Therefore, we firstly predicted that positive results in the gene screening of NRPS and PKS-II genes could provide the evidences of the corresponding metabolites. However, it is known that genome-sequencing projects on different actinobacteria have revealed a large number of biosynthetic gene clusters in each genome [44]. This viewpoint is proved by the genome information of *Streptomyces peucetius* ATCC 27952, possessing 68 biosynthetic gene clusters (BCGs) for various types of secondary metabolites, including non-ribosomal peptide synthase (NRPS), polyketide synthase (PKS I, II, and III), terpenes, and others [45]. Hence, the chosen primer system used in this work, in spite of favorable for the great majority of known PKS/NRPS genes, may not work in all cases of polyketides and nonribosomal peptides with uncommon molecular constructions. For example, strain SYP-A7257 exhibiting no NRPS amplicons produced actinomycin derivatives synthesized via a NRPS biosynthetic gene cluster [46, 47]. Meanwhile, the detected NRPS or PKS gene fragments may not clearly assure the presence of the corresponding metabolites. In the study of Schneemann et al. (2010) [43], 10 of the strains yielded PCR products with the PKS-specific primers, but no corresponding PKS products were detected under the laboratory conditions. Within our study, strain SYP-A7257 produced compounds **F1** (fungichromin) and **F2** (actinomycin derivates) synthesized via PKS and NRPS gene clusters [46, 47], respectively, which did not match the obtained PKS genes involved in biosynthesis of meridamycin (PKSI) [28] in the functional gene screening. It is possible that the detected biosynthetic gene clusters are cryptic under laboratory condition, which leads to a low efficiency in the discovery of their corresponding metabolites [44]. It is also possible that the detected biosynthetic gene clusters are nonfunctional. Nevertheless, the PCR-based prescreening of isolates with the primers targeting on genes involving in the biosynthesis of secondary metabolites is an effective approach for detecting useful bioactive compounds [43, 48, 49].

To our knowledge, the genes encoding PKS and NRPS systems from these *Streptomyces* isolates might both play a key role in producing secondary metabolites with antagonistic activities against plant pathogenic microorganisms [50]. This opinion was similar to the studies of Sharma et al. (2016) [51] and Passari et al. (2015) [52], which demonstrated that streptomycetes having antimicrobial activities were positive for the presence of the genes related to these two biosynthetic pathways in their genomes because genes encoding PKS-I and NRPS might be responsible for both of their involvement in the regulation of antimicrobial activity in microorganisms. Within the present study, the presence of the biosynthetic genes PKS and NRPS in the rhizospheric streptomycetes isolates related to *P. notoginseng* suggests that these beneficial strains may be the important producers of bioactive secondary metabolites. For example, strain SYP-A7257 having a positive hit in PKS gene screening produced eight antagonistic compounds consisting of one so far unidentified compound, among which four isolated compounds exhibited favorable activities against the test bacteria and fungi. Meanwhile, compounds **F1** and **F2** both displayed antagonistic activities to the plant pathogenic bacteria (*A. marplatensis*) with MIC values of 14.6 and 49.4 μg/ml, respectively. These results indicate that these rhizospheric streptomycetes may be good candidates for biocontrol agents of *P. notoginseng* because immense diversity of PKS genes detected could indicate a diversity of antimicrobial secondary metabolites.

## Conclusion

In summary, this research demonstrates the distribution and diversity of the *P. notoginseng* rhizospheric soil-derived streptomycetes in respect to their metabolic potential for polyketides and non-ribosomal peptides. Many questions remain in respect to the ecological function of *Streptomyces* strains in the rhizospheric environment, their evolution and biogeographic distribution. However, a diversity of streptomycetes and their potential to produce bioactive secondary metabolites suggested that these *Streptomyces* isolates might represent a valuable resource of bioactive secondary metabolites with antimicrobial activities and genes encoding their biosynthesis. That potential should not be overlooked. Meanwhile, the farm experiments indicated that *Streptomyces* sp. SYP-A7257 can be used as a promising biocontrol agent for promoting the healthy growth of *P. notoginseng* due to the chemical defense substances produced from this strain.

## Methods

### Soil sample collection and isolation of actinobacteria strains

Twelve rhizospheric soil samples in the cultivation field of *P. notoginseng* were collected in the Wenshan region of Yunnan Province (Table S1). After removing the most amount of soil, the residue rhizospheric soil was gently stripped and collected around 1 cm from the main roots. Three parallel soil samples at each site were mixed to obtain one sample. All the soil samples were sieved (< 4 mm) to remove stones and plant residues and then were placed into sterile bags. Finally, they were kept at 4 °C before treatment.

Actinobacteria were isolated by using serial dilution spread plate method. Five grams of soil was mixed with 45 ml of sterile 0.9% NaCl and stirred for 1 h at 25 °C. The soil suspension stood for 10 min at room temperature to allow precipitation of large particles. Serial 10-fold dilutions (from 10^− 3^ to 10^− 5^) were made with 0.9% NaCl. 100 μl of diluents were transferred to Petri dishes with 38^#^ agar medium (4.0 g yeast extract, 2.0 g malt flour, 4.0 g glucose, 1.0 ml trace elements solution, a few vitamins complex containing 0.001 g riboflavin, 0.001 g nicotinic acid, 0.001 g creatinol, 0.001 g calcium pantothenate, 0.001 g biotin, 0.001 g p-aminobenzoic acid, 0.001 g vitamin B1, 0.001 g vitamin B6, 20.0 g agar and 1.0 L distilled water, pH 7.2) supplemented with 100 mg/L nystatin. Trace elements solution contained 1.0 g FeSO_4_•7H_2_O, 1.0 g MnCl_2_•4H_2_O, 1.0 g ZnSO_4_•7H_2_O and 1.0 L distilled water. Then, the plates were incubated at 28 °C for 1–4 week, and all well separated actinobacterial colonies were picked from the original isolation plate and repeatedly sub-cultured on 38^#^ agar medium until pure cultures were obtained as judged by uniform colony morphology. The purified strain was preserved as a spore suspension in 25% glycerol at − 80 °C. Totally 42 of purified isolates were obtained.

### DNA extraction, sequencing and analysis

The genomic DNA from the pure culture of each isolate was extracted by using a method of Muyzer et al (1993) [24]. The 16S rRNA genes were amplified by using the universal primer pairs (Table S2) [24], and then PCR products were subjected to gene sequencing by Sangon Biotech (Shanghai, China). The 16S rRNA gene sequences were compared with the EzTaxon-e databases by using the Basic Local Alignment Search Tool (nucleotide blast) (https://blast.ncbi.nlm.nih.gov/Blast.cgi).

### Detection of PKSI, PKSII and NRPS genes

Three sets of degenerate primers targeting on genes encoding PKS I, PKS II and NRPS were performed as recommended in Table S2. Thermocycling conditions were similar to the method described by Li et al (2014) [14]. All correctly sized PCR products were immediately cloned by using the *pEASY*®-T5 Zero cloning kit (Transgen Co., China) based on the manufacturer’s suggested protocol. Plasmids were isolated and subjected to gene sequencing by Sangon Biotech (Shanghai, China). All sequences were compared with GenBank databases by using BlastX and were analyzed to construct the phylogenetic tree by the neighbor-joining method [53] using the MEGA software package, version 7.0. The stability of relationships was evaluated by performing bootstrap at 1000 resamplings [54].

### Nucleotide sequence accession numbers

The 16S rRNA gene sequences of 42 isolates have been deposited in GenBank under the accession numbers given in Table S3. Accession numbers for PKSI, PKSII and NRPS gene sequences were assigned as follows: MH151921, MH151922, MH198457-MH198523, MH198525- MH198530 (Table S4).

### Fermentation, extraction and HPLC-DAD analysis of culture extracts

Each isolate was grown in a 300-ml Erlenmeyer flask containing 100 ml fermentation broth at 28 °C with shaking at 250 rpm in the dark. Two media were employed to cultivate these strains, which included GYM4 [43] and SPM1 (40.0 g soluble starch, 5.0 g glucose, 25.0 g soybean meal, 5.0 g yeast extract, 0.5 g K_2_HPO_4_, 0.5 g MgSO_4_, 1.0 g CaCO_3_, 1.0 L distilled water, pH 7.5). After 5 to 7 days of cultivation, the fermentation broth was extracted with equal volume of ethyl acetate (EtOAc). Each EtOAc extract was then evaporated under reduced pressure to yield a crude extract. Subsequently, each EtOAc extract was dissolved in methanol (MeOH) for HPLC-DAD analysis on an Agilent 1260 series (Agilent Technologies, USA) with a Diode Array Detector (DAD) (200–600 nm) and a C18 RP-column (Platisil ODS-C18 5 μm, 4.6 × 250 mm), with a gradient from 10% acetonitrile in water to 100% acetonitrile over 60 min. The EtOAc extract of strain SYP-A7257 was further subjected to mass spectrometry by Agilent HPLC-HRMS system (Agilent 1260/6530 LC-QTOF/MS, USA) equipped with an Agilent ZORBAX SB-C18 column (4.6 × 50 mm, 1.8 μm) at 40 °C. Mass spectra data with the scanning range from 100 to 2000 amu were collected in the positive and negative modes. Compounds were identified by comparing molecular weights, UV spectra, and retention times with published chemical data from references and standard databases, such as Dictionary of Natural Products on DVD, version 22.2, and SciFinder 2007.

### Isolation and identification of antimicrobial compounds

To obtain antimicrobial compounds, the fermentation study (3 L) was performed on targeting SYP-A7257, which were selected on the basis of functional gene screening, antimicrobial activity and chemical analysis. The fermentation of strain SYP-A7257 was carried out in SPM1 medium. Cultures were incubated at 28 °C for 5 days on rotary shakers at 300 rpm in the dark.

To obtain antimicrobial compounds of strain SYP-A7257, the mycelium and cultural liquid broth were separated by centrifugation at 6000 rpm. Metabolites were extracted from the supernatant with 3 L of ethyl acetate and from the mycelium with 500 ml MeOH. The extracts from mycelium and supernatant were combined and evaporated to obtain the crude sample (27 g), further fractioned by silica gel column chromatography using chloroform-methanol gradient elution to yield four fractions (A-D). Fraction A (0.8 g) was separated again by Sephadex LH-20 column chromatography using MeOH as eluant, and the resulting subfractions were combined and further purified by semi-preparative HPLC (CH_3_CN-H_2_O, 60%) to afford compound **F1** (6.1 mg). Fraction D (0.6 g) was charged on ODS-C18 reverse chromatography column using MeOH-H_2_O gradient elution to give five subfractions (D1-D5). Subfraction D3 (60 mg) was further purified by semi-preparative HPLC (CH_3_CN-H_2_O, 35%) to afford compound **F2** (9.4 mg).

Scale-up cultivation for strain SYP-A7257 was performed two times in a 5-L stirred fermentation tank in order to attain compounds **F7** and **F8** at a fermentation time of 96 h. For isolation, a 10-L aliquot of the culture filtrate were extracted with EtOAc to get the crude extract (30.0 g) and purified by chromatography on a silica gel column with chloroform-MeOH gradient elution, yielding five fractions (E-I). Fraction I (1.4 g) was subjected to chromatography on ODS-C18 reverse column using MeOH-H_2_O gradient to yield five subfractions (I1-I5). Fraction I3 (517.9 mg) was further purified by Sephadex LH-20 column chromatography using MeOH as eluant followed by preparative reversed-phase HPLC to afford compound **F8** (2.0 mg). Fraction I5 (50mg) was subjected to preparative reversed-phase HPLC to give compound **F7** (2.2 mg).

The NMR spectra were run on Bruker AVANCE-600 NMR spectrometers (Rheinstetten, Germany). The chemical shifts were expressed in *d* (ppm) using CDCl_3_, CD_3_OD and DMSO-*d*_6_ as solvent and TMS as internal reference. ESI-MS was recorded on an Aligent Quattro Premier Triple Quadrupole mass spectrometer. Semi-preparative HPLC was carried out on SHIMADZU 20A-DAD HPLC using Welch Materials Ultimate XB-C18 (5 μm) 10 × 250 mm column. Size exclusion chromatography was done on Sephadex LH-20 (Amersham, GE in USA).

### Evaluation of antimicrobial activity

The extracts and compounds were dissolved in DMSO for antimicrobial activity assay using the 96-well flat-bottomed method [27] against human pathogenic bacteria (*Staphylococcus aureus*, *Escherichia coli*, *Enterococcus faecium*, *Proteus mirabilis* and *Acinetobacter baumannii*), human pathogenic fungi (*Candida albicans*) and *P. notoginseng* pathogenic bacteria (*Achromobacter marplatensis*). Disk diffusion method [55] was used to test the substance activity against the mycelial pathogenic fungus (*Fusarium solani*) of *P. notoginseng*. All these pathogens were stored in School of Life Science and Biopharmaceutics, Shenyang Pharmaceutical University, China. The bacterial suspension was diluted to the initial concentration (optical density of 0.3 at 600 nm; OD_600_ = 0.3). A 500 μl bacterial solution was added into 50 ml LB media. The bacterial solution was transferred into a 96-well plate (198 μl per well). Then, 2 μl of the extracts (50 g L^− 1^) or compounds (50 mg L^− 1^) dissolved in DMSO was added into a 96-well plate in order to reach the final volume of 200 μl per well. Here, untreated bacterial solution was considered as the negative control group. Bacterial solution treated with vancomycin served as and positive control group. The growth was measured by using a multiple photometric reader after 24 h of incubation at 37 °C. Every experiment was performed in triplicate. For inhibition test against mycelial pathogenic fungus, a 25 μl volume of the extract suspension was pipetted onto each disk (diameter, 7 mm) and incubated for 96 h at 28 °C in the dark before the inhibition zone measurement. A 25 μl volume DMSO was used as negative control. Geneticin (G418) served as positive control.

Actinomycin X_2_ (**F1**), fungichromin (**F2**), thailandin B (**F7**) and antifungalmycin (**F8**) were analyzed by the above same method to determine the MICs (Minimum Inhibitory Concentration) against all the tested microorganisms.

### The farm experiments

The farm land is located at Bazi *P. notoginseng* plantation (104°6′12.9″E, 23°49′46.9″N) in Wenshan, Yunnan province, which is the main *P. notoginseng* producing region in China. The healthy *P. notoginseng* seedlings were harvested last winter. The field was covered with 2.5-m high shade shelter, which had a good ventilated condition. Chemical fumigants as described by Gao et al [56] was applied to treat the soil prior to replanting *P. notoginseng*. The land was divided into 6 plots on the basis of a completely random block design, three plots as treatment group and another three plots as control group. 400 of 1-year-old healthy *P. notoginseng* seedlings were planted in each plot (1.2 × 3.0 m) according to the method of Sun et al [57]. A 10-L of 1:200 diluent broth of strain SYP-A7257 fermented in SPM1 medium at 28 °C for 5 days was applied to the soil by root irrigation [58] in the treatment (T) group. Meanwhile, SPM medium dilution without strain cultivation was applied to the soil by root irrigation in the control (CK) group. The root irrigation experiments were continually carried out 3 times at 2, 3 and 7 months after the emergence of the *P. notoginseng* seedlings. Seedling emergence and dead seedling rate were recorded every 30 days after the first root irrigation. The survival rate (%) of each plot was calculated using the following equation: The survival rate (%) = (the number of survival seedlings/400) × 100.

### Statistical analysis

The statistical analysis was performed by using Graph Pad Prism 5.01. All the data were present as means± SD of triplicate experiments. One-way analysis of variance (ANOVA) followed by Turkey’s test was used to express the statistical differences between groups. The value of *P* < 0.05 was presented as statistically significant.

## Supplementary information

**Additional file 1 Figure S1**. HPLC-DAD analysis of crude extracts of SYP-A7257 cultured in SYM1 medium. Extracts were analyzed using Agilent 1260 series (Agilent Technologies, USA) with a Diode Array Detector (DAD) (200–600 nm) and a C18 RP-column (Platisil ODS-C18 5 μm, 4.6 × 250 mm), with a gradient from 10% acetonitrile in water to 100% acetonitrile over 60 min., 1.0 ml/min as the flow rate. **Figure S2.** TIC chromatogram of the extract of strain SYP-A 7257 and MS spectra of peaks F1–F6 obtained by HPLC–HRESIMS in positive mode. **Figure S3.** HR**-**ESI-MS in positive-ion mode and UV/vis characteristics of compound F1. **Figure S4.** HR**-**ESI-MS in positive-ion mode and UV/vis characteristics of compound F2. **Figure S5.** HR**-**ESI-MS in positive-ion mode and UV/vis characteristics of compound F3. **Figure S6.** HR**-**ESI-MS in positive-ion mode and UV/vis characteristics of compound F4. **Figure S7.** HR**-**ESI-MS in positive-ion mode and UV/vis characteristics of compound F5. **Figure S8.** HR**-**ESI-MS in positive-ion mode and UV/vis characteristics of compound F6. **Figure S9.** ESI-MS in positive and negative-ion mode of compound F1. **Figure S10.**^1^H NMR spectrum of compound F1 in CDCl_3_ recorded at 600 MHz. **Figure S11.**^13^C NMR spectrum of compound F1 in CDCl_3_ recorded at 150 MHz. **Figure S12.** ESI-MS in positive and negative-ion mode of compound F2. **Figure S13.**^1^H NMR spectrum of compound F2 in CD_3_OD recorded at 600 MHz. **Figure S14.**^13^C NMR spectrum of compound F2 in CD_3_OD recorded at 150 MHz. **Figure S15.** ESI-MS in negative-ion mode of compound F7. **Figure S16.**^1^H NMR spectrum of compound F7 in CD_3_OD recorded at 600 MHz. **Figure S17.**^13^C NMR spectrum of compound F7 in CD_3_OD recorded at 150 MHz. **Figure S18.** ESI-MS in positive-ion mode of compound F8. **Figure S19.**^1^H NMR spectrum of compound F8 in CD_3_OD recorded at 600 MHz. **Figure S20.**^13^C NMR spectrum of compound F8 in CD_3_OD recorded at 150 MHz. **Table S1.** Characteristics of rhizospheric soil samples of *P. notoginseng* in the Wenshan region of Yunnan Province, China. **Table S2.** PCR primers used in this study. **Table S3.***Streptomyces* isolated from different soil of *P. notoginseng* with similarity values of 16S rRNA gene sequences to the closest cultivated species. **Table S4.** KS domain and NRPS amino acid sequences of the soil-derived *Streptomyces* isolates from the rhizospheric soil of *P. notoginseng*. **Table S5.** NMR Data for compound F1 (600 MHz) in CDCl_3_ (δ in ppm, J in Hz). **Table S6.** NMR Data for compound F2 (600 MHz) in CD_3_OD (δ in ppm, J in Hz). **Table S7.** NMR Data for compound F7and F8 (600 MHz) in CD_3_OD (δ in ppm, J in Hz).

## Data Availability

All data generated or analyzed during this study are included in this published article and its supplementary information files.

## Abbreviations

PKS I: Type I polyketide synthases
PKS II: Type II polyketide synthase
NRPS: Nonribosomal peptide synthetases
HPLC-DAD: High Performance Liquid Chromatography-Diode Array Detector
HPLC-HRMS: High Performance Liquid Chromatography-High Resolution Mass Spectrometry
UV: Ultraviolet
NMR spectra: Nuclear magnetic resonance spectra
TIC: Total ion current
BCGs: Biosynthetic gene clusters
Blast: Basic Local Alignment Search Tool
EtOAc: Ethyl acetate
MeOH: Methanol
ANOVA: One-way analysis of variance
