# Supplementary material for: Insights into Streptomyces spp. isolated from the rhizospheric soil of Panax notoginseng: isolation, antimicrobial activity and biosynthetic potential for polyketides and non-ribosomal peptides
Source: BMC Microbiol. 2020 Jun 3;20:143. doi: 10.1186/s12866-020-01832-5 (PMC7271549; doi:10.1186/s12866-020-01832-5)
Supplement: Supplementary file 1 — Additional file 1 Figure S1. HPLC-DAD analysis of crude extracts of SYP-A7257 cultured in SYM1 medium. Extracts were analyzed using Agilent 1260 series (Agilent Technologies, USA) with a Diode Array Detector (DAD) (200–600 nm) and a C18 RP-column (Platisil ODS-C18 5 μm, 4.6 × 250 mm), with a gradient from 10% acetonitrile in water to 100% acetonitrile over 60 min., 1.0 ml/min as the flow rate. Figure S2. TIC chromatogram of the extract of strain SYP-A 7257 and MS spectra of peaks F1–F6 obtained by HPLC–HRESIMS in positive mode. Figure S3. HR-ESI-MS in positive-ion mode and UV/vis characteristics of compound F1. Figure S4. HR-ESI-MS in positive-ion mode and UV/vis characteristics of compound F2. Figure S5. HR-ESI-MS in positive-ion mode and UV/vis characteristics of compound F3. Figure S6. HR-ESI-MS in positive-ion mode and UV/vis characteristics of compound F4. Figure S7. HR-ESI-MS in positive-ion mode and UV/vis characteristics of compound F5. Figure S8. HR-ESI-MS in positive-ion mode and UV/vis characteristics of compound F6. Figure S9. ESI-MS in positive and negative-ion mode of compound F1. Figure S10.1H NMR spectrum of compound F1 in CDCl3 recorded at 600 MHz. Figure S11.13C NMR spectrum of compound F1 in CDCl3 recorded at 150 MHz. Figure S12. ESI-MS in positive and negative-ion mode of compound F2. Figure S13.1H NMR spectrum of compound F2 in CD3OD recorded at 600 MHz. Figure S14.13C NMR spectrum of compound F2 in CD3OD recorded at 150 MHz. Figure S15. ESI-MS in negative-ion mode of compound F7. Figure S16.1H NMR spectrum of compound F7 in CD3OD recorded at 600 MHz. Figure S17.13C NMR spectrum of compound F7 in CD3OD recorded at 150 MHz. Figure S18. ESI-MS in positive-ion mode of compound F8. Figure S19.1H NMR spectrum of compound F8 in CD3OD recorded at 600 MHz. Figure S20.13C NMR spectrum of compound F8 in CD3OD recorded at 150 MHz. Table S1. Characteristics of rhizospheric soil samples of P. notoginseng in the Wenshan region of Yunnan Province, China. Table [file 12866_2020_1832_MOESM1_ESM.doc]

**Supplementary information**

**Insights into *Streptomyces* isolated from the rhizospheric soil of** ***Panax notoginseng*: isolation, antimicrobial activity and biosynthetic potential for polyketide and non-ribosomal peptide**

Fei Peng^1,2§^, Meng-Yue Zhang^1§^, Shao-Yang Hou^1^, Juan Chen^1^, Ying-Ying Wu^1^, Yi-Xuan Zhang^1^*

1.School of Life Science and Biopharmaceutics, Shenyang Pharmaceutical University, Shenyang, PR China

2.Quanzhou Medical college, Quanzhou, PR China

^§^Fei Peng and Meng-Yue Zhang are Co-first authors

* Corresponding author: Yi-Xuan Zhang, School of Life Science and Biopharmaceutics, Shenyang Pharmaceutical University, Shenyang 110016, PR China.

E-mail addresses: [zhangyxzsh@163.com](mailto:zhangyxzsh@163.com).

**Figure S1** HPLC-DAD analysis of crude extracts of SYP-A7257 cultured in SYM1 medium. Extracts were analyzed using Agilent 1260 series (Agilent Technologies, USA) with a Diode Array Detector (DAD) (200−600 nm) and a C18 RP-column (Platisil ODS-C18 5 μm, 4.6 × 250mm), with a gradient from 10% acetonitrile in water to 100% acetonitrile over 60 min., 1.0 ml/min as the flow rate.

**Figure S2** TIC chromatogram of the extract of strain SYP-A 7257 and MS spectra of peaks **F1–F6** obtained by HPLC–HRESIMS in positive mode.

**Figure S3** HR**-**ESI-MS in positive-ion mode and UV/vis characteristics of compound **F1**

**Figure S4** HR**-**ESI-MS in positive-ion mode and UV/vis characteristics of compound **F2**

**Figure S5** HR**-**ESI-MS in positive-ion mode and UV/vis characteristics of compound **F3**

**Figure S6** HR**-**ESI-MS in positive-ion mode and UV/vis characteristics of compound **F4**

**Figure S7** HR**-**ESI-MS in positive-ion mode and UV/vis characteristics of compound **F5**

**Figure S8** HR**-**ESI-MS in positive-ion mode and UV/vis characteristics of compound **F6**

**Figure S9** ESI-MS in positive and negative-ion mode of compound **F1**

**Figure S10** ^1^H NMR spectrum of compound **F1** in CDCl_3_ recorded at 600 MHz.

**Figure S11** ^13^C NMR spectrum of compound **F1** in CDCl_3_ recorded at 150 MHz

**Figure S12** ESI-MS in positive and negative-ion mode of compound **F2**

**Figure S13** ^1^H NMR spectrum of compound **F2** in CD_3_OD recorded at 600 MHz.

**Figure S14** ^13^C NMR spectrum of compound **F2** in CD_3_OD recorded at 150 MHz

**Figure S15** ESI-MS in negative-ion mode of compound **F7**

**Figure S16** ^1^H NMR spectrum of compound **F7** in CD_3_OD recorded at 600 MHz.

**Figure S17** ^13^C NMR spectrum of compound **F7** in CD_3_OD recorded at 150 MHz.

**Figure S18** ESI-MS in positive-ion mode of compound **F8**

**Figure S19** ^1^H NMR spectrum of compound **F8** in CD_3_OD recorded at 600 MHz.

**Figure S20** ^13^C NMR spectrum of compound **F8** in CD_3_OD recorded at 150 MHz.

**Table S1** Characteristics of rhizospheric soil samples of *P. notoginseng* in the Wenshan region of Yunnan Province, China

**Table S2** PCR primers used in this study

**Table S3** *Streptomyces* isolated from different soil of *P. notoginseng* with similarity values of 16S rRNA gene sequences to the closest cultivated species

**Table S4** KS domain and NRPS amino acid sequences of the soil-derived *Streptomyces* isolates from the rhizospheric soil of *P. notoginseng*.

**Table S5** NMR Data for compound **F1** (600 MHz) in CDCl_3_ (δ in ppm, J in Hz)

**Table S6** NMR Data for compound **F2** (600 MHz) in CD_3_OD (δ in ppm, J in Hz)

**Table S7** NMR Data for compound **F7**and **F8** (600 MHz) in CD_3_OD (δ in ppm, J in Hz)

**Figure S1**

**mAU**


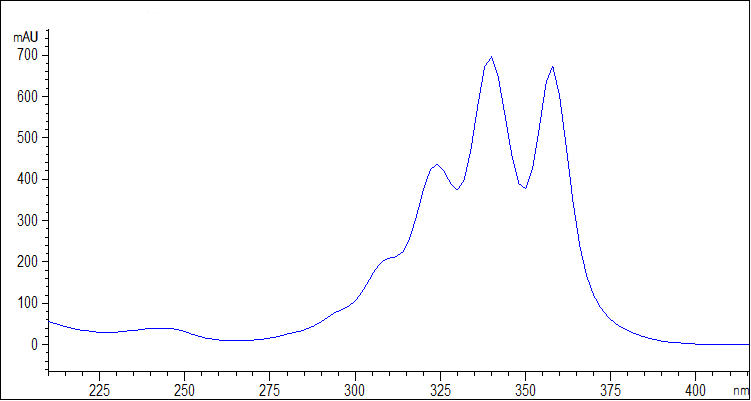

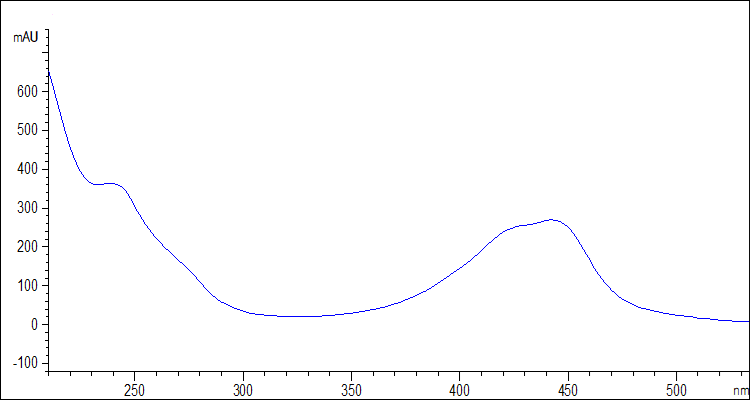

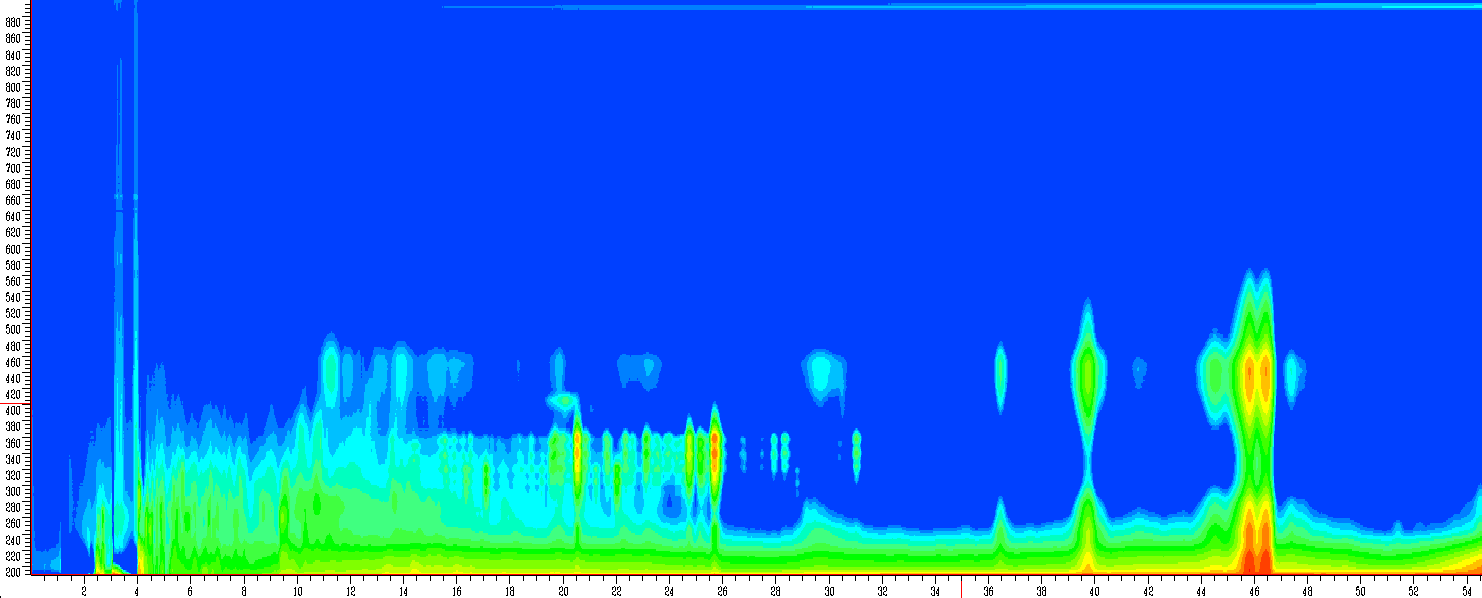


**min**

**325**

**330**

**360**

**Target peak 1**

**446**

**241**

**Target peak 2**

**Figure S2**

**F5**

**F6**

**F2**

**F3**

**F1**

**F4**

**min**

**Figure S3**

**
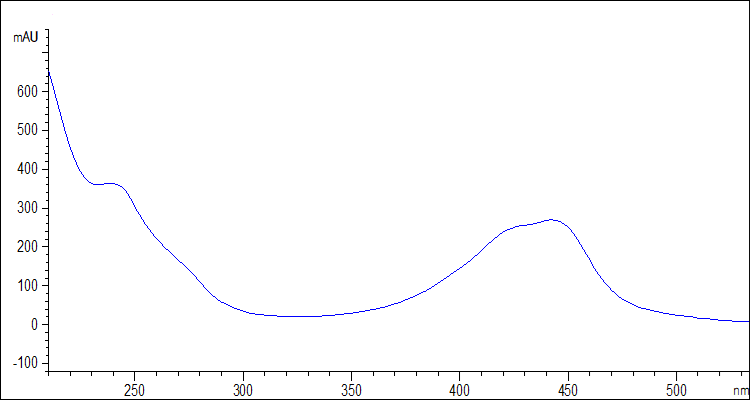
**

**Figure S4**

**
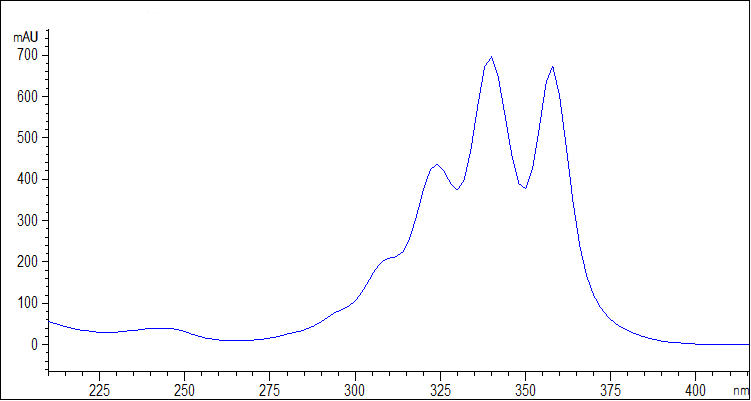
**

**Figure S5**

**
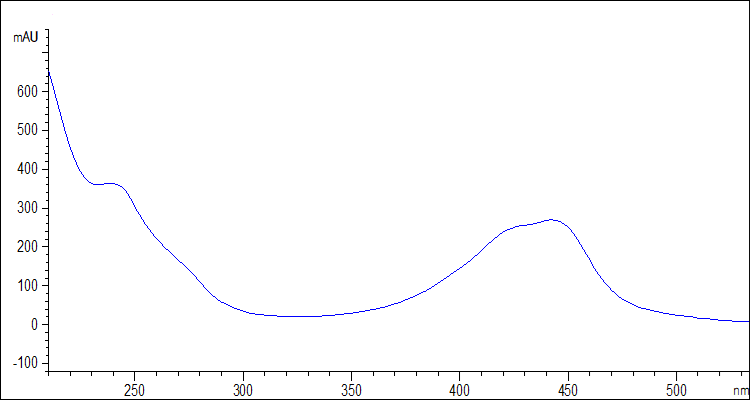
**

**Figure S6**

**
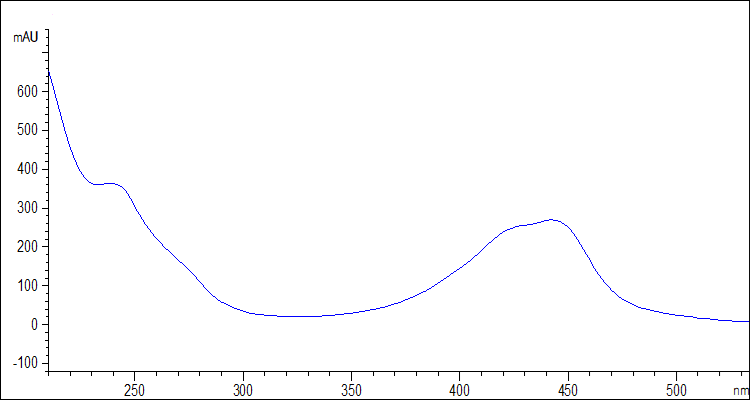
**

**Figure S7**

**
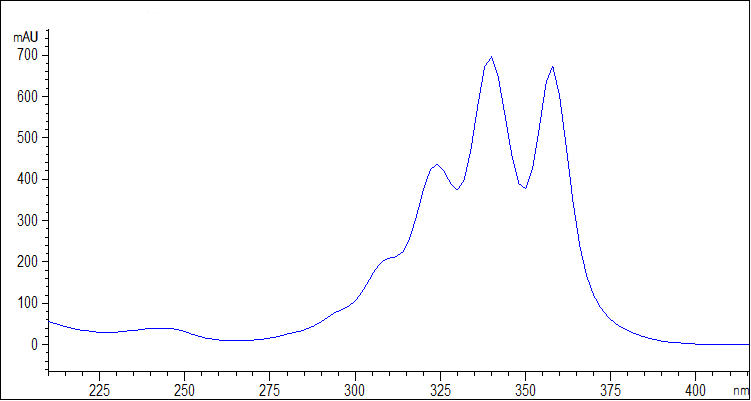
**

**Figure S8**

**
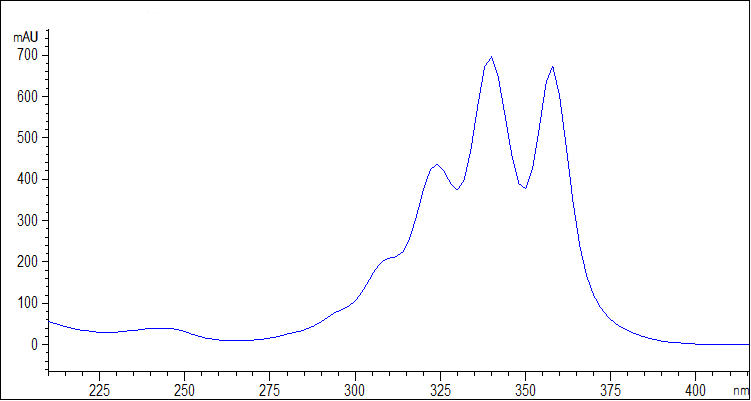
**

**Figure S9**


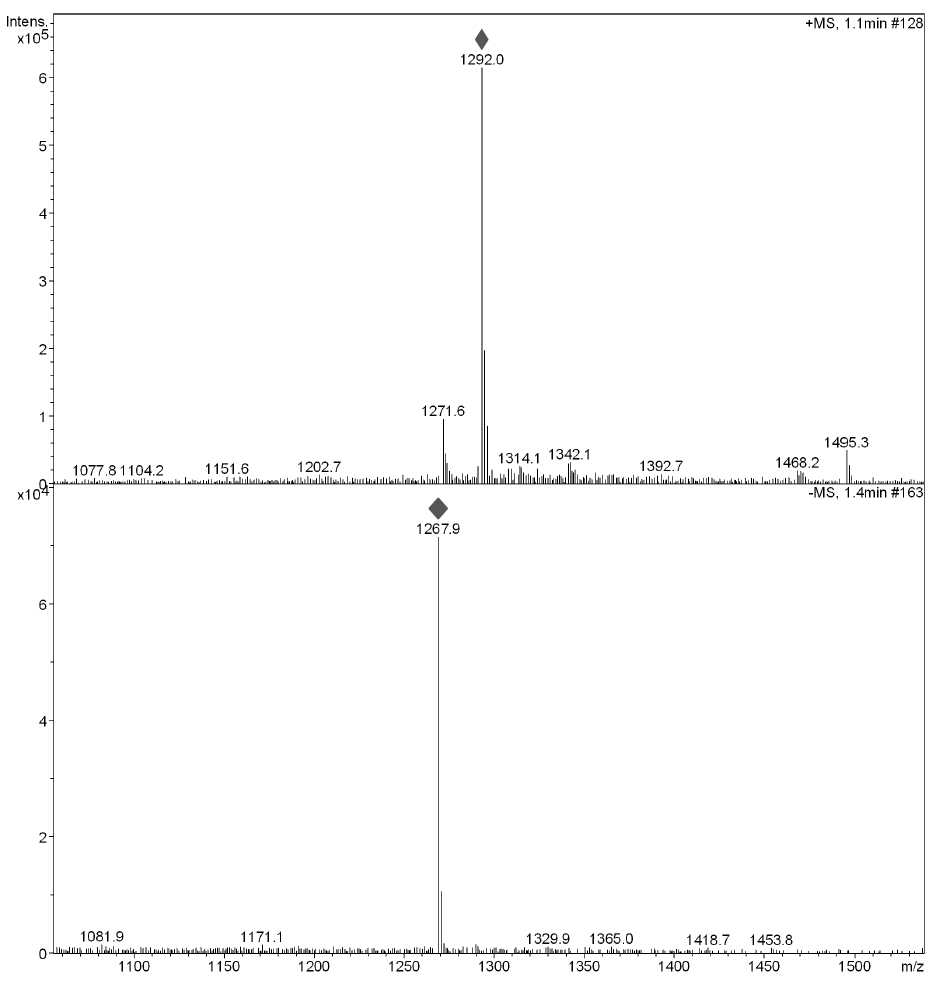


**Figure S10**.

**
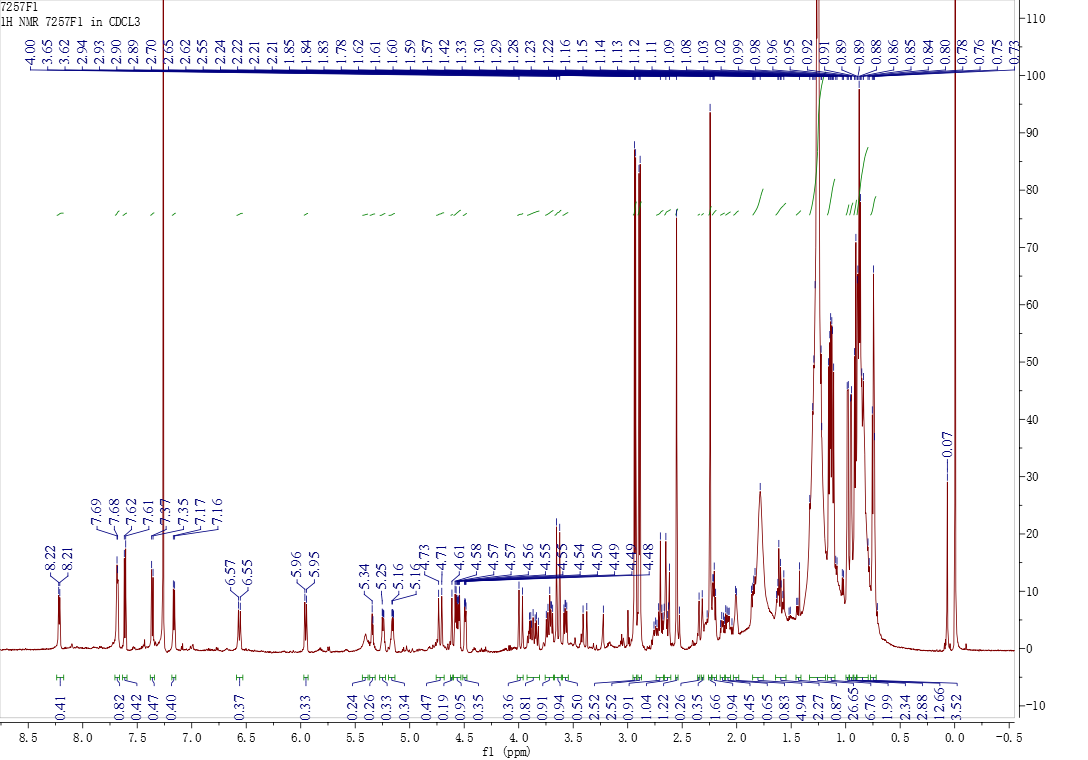
**

**Figure S11
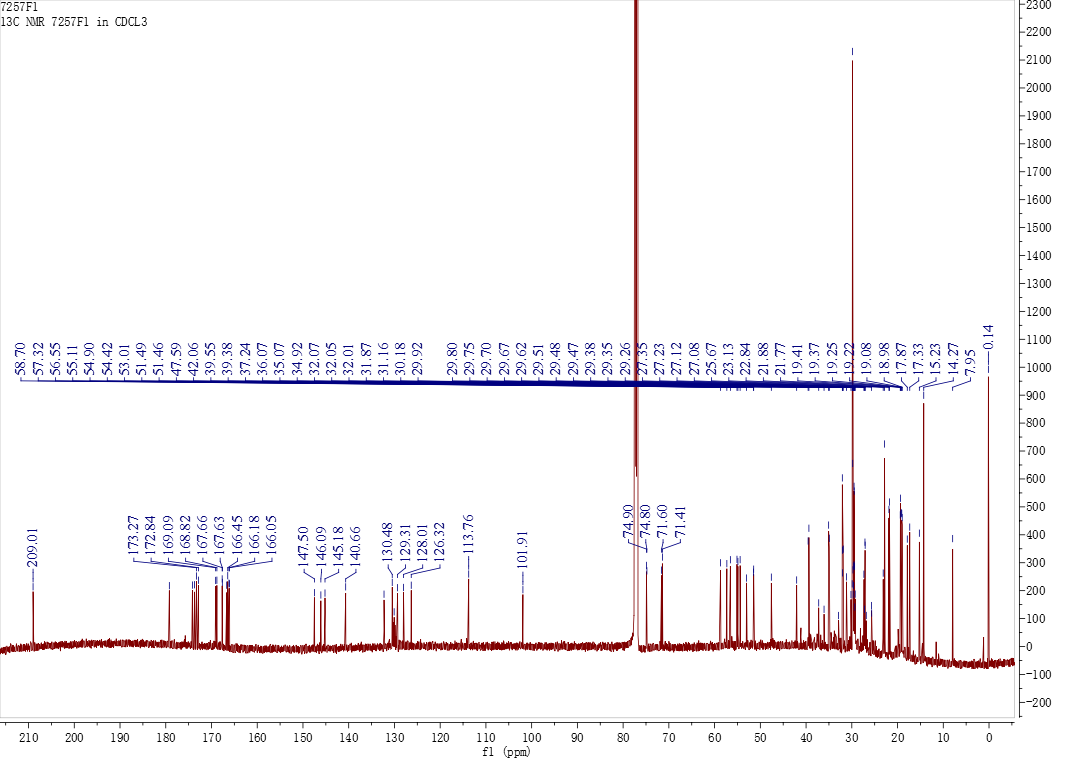
**

**Figure S12**
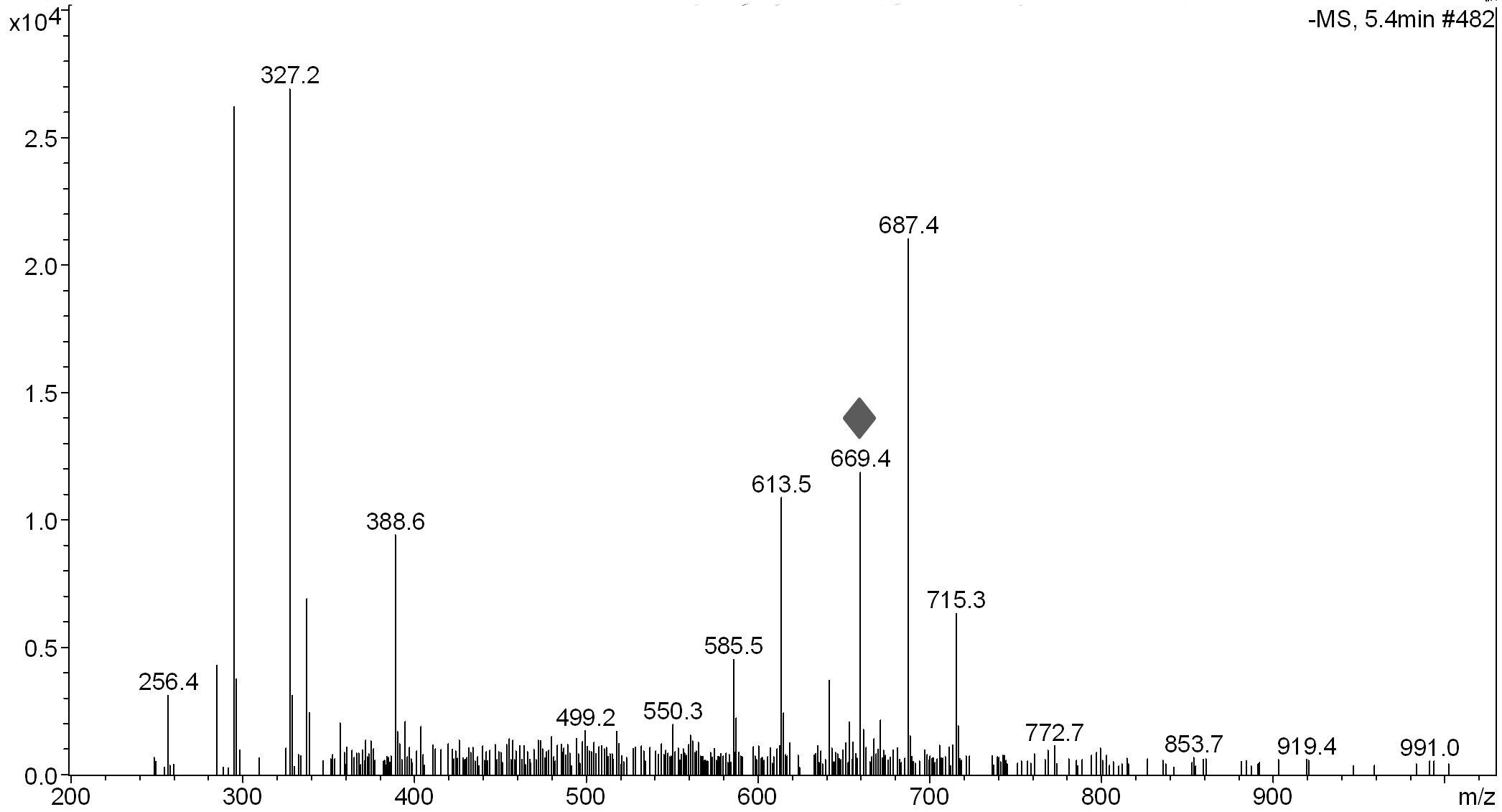


**Figure S13**

**
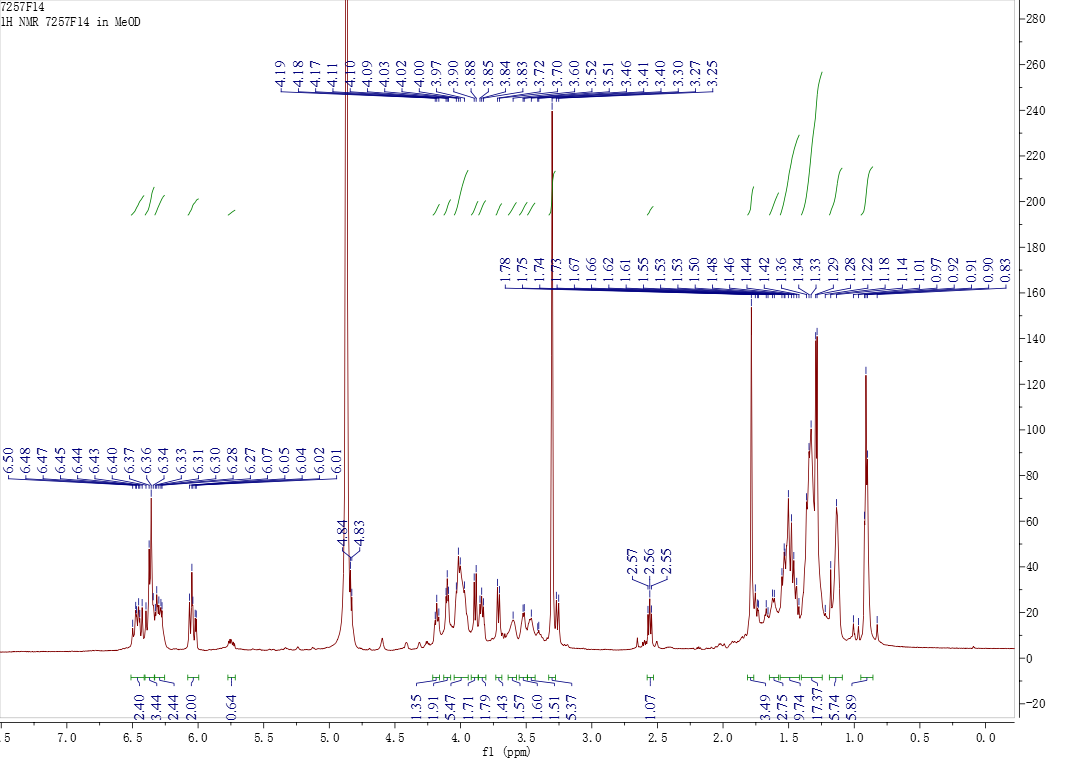
**

**Figure S14**

**
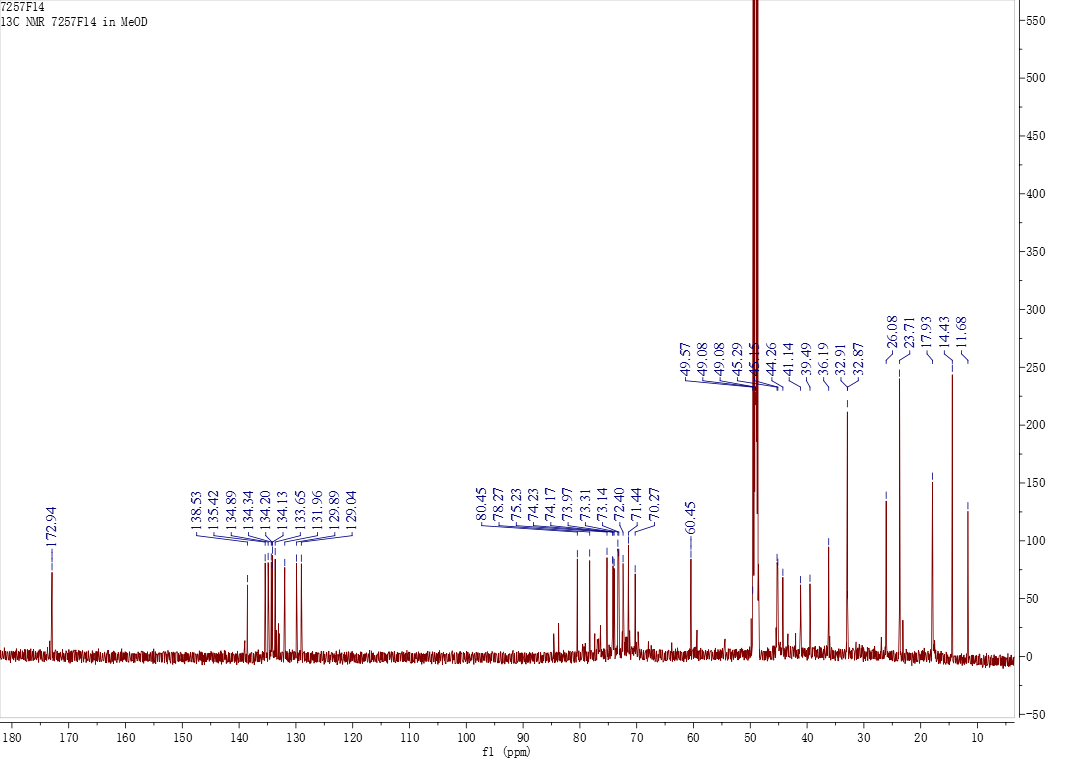
**

**Figure S15**


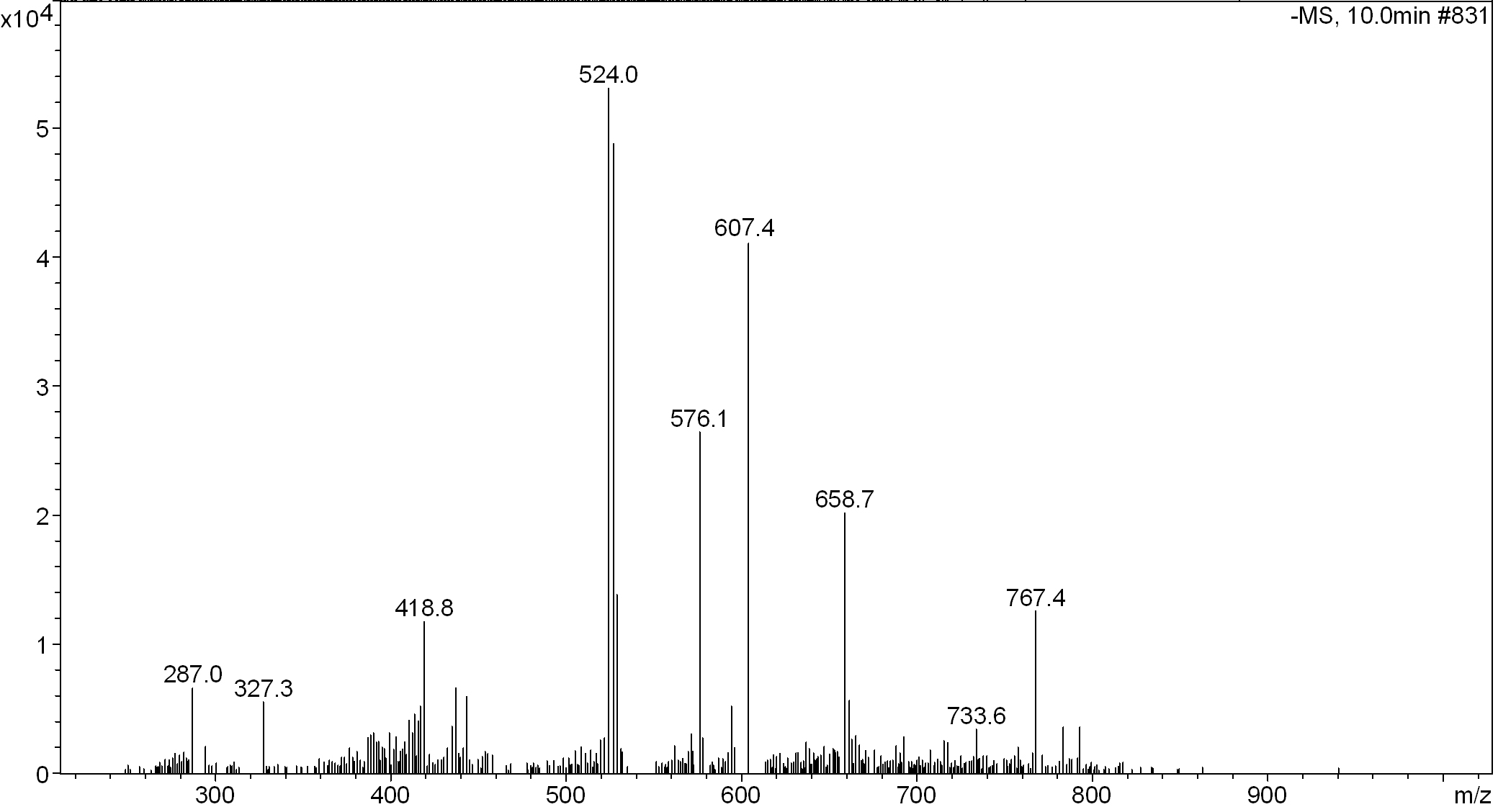


**Figure S16**

**
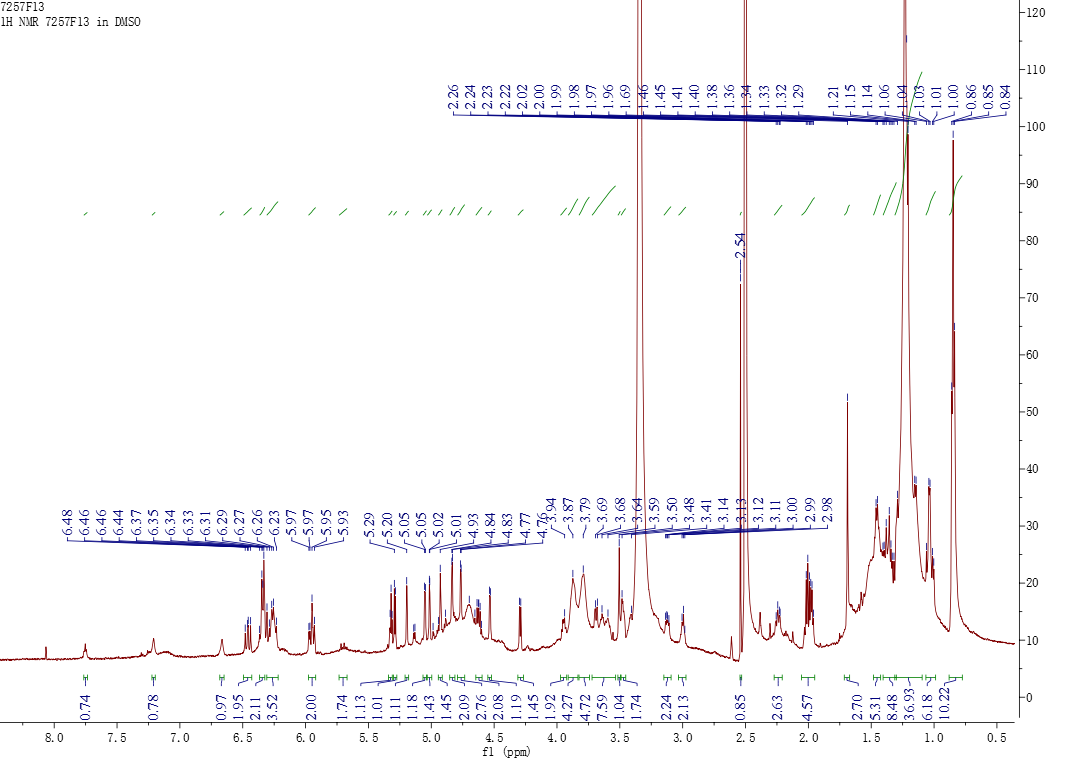
**

**Figure S17**

**
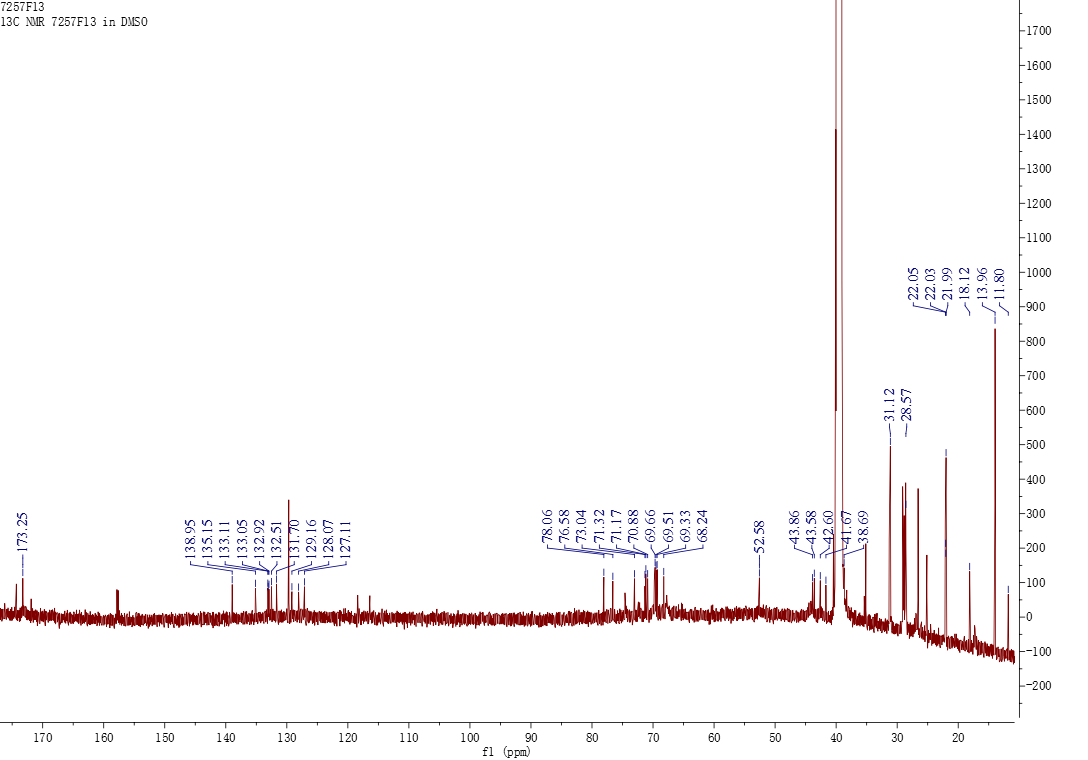
**

**Figure S18**


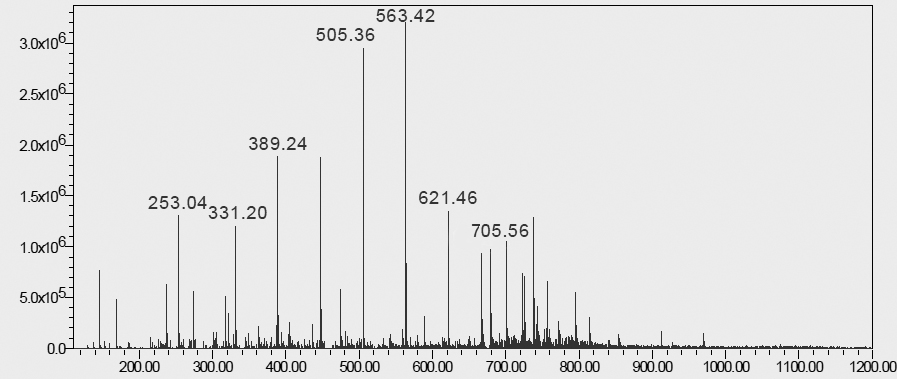


**Figure S19**

**
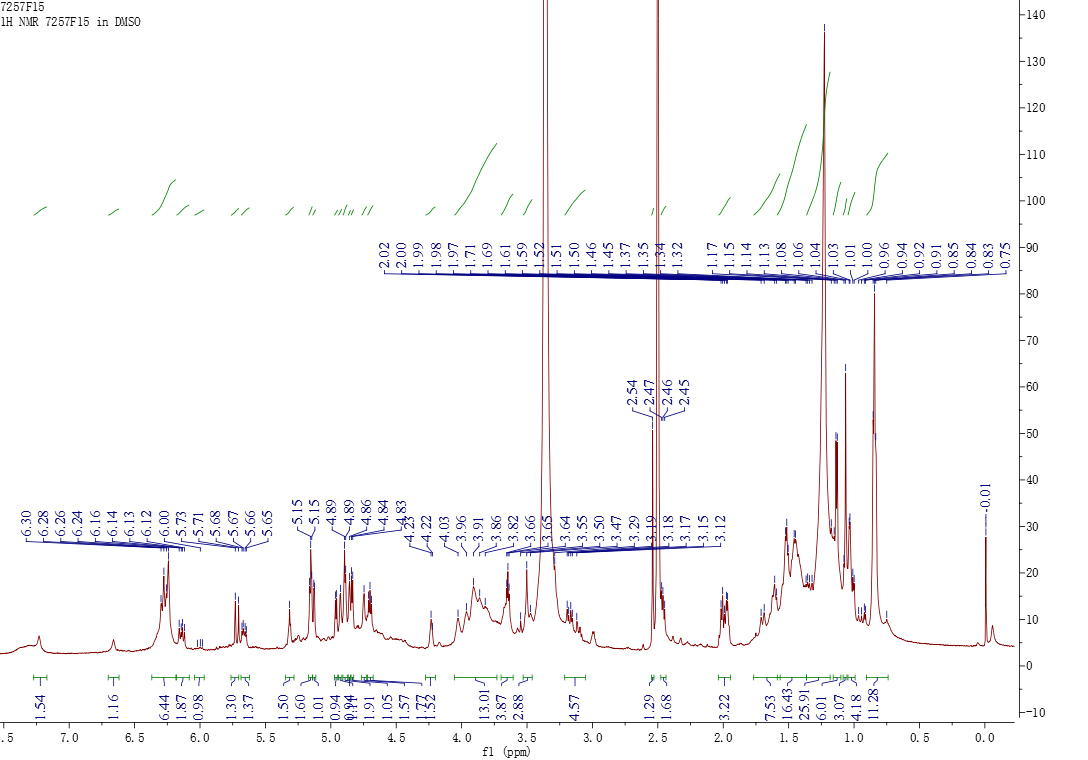
**

**Figure S20**

**
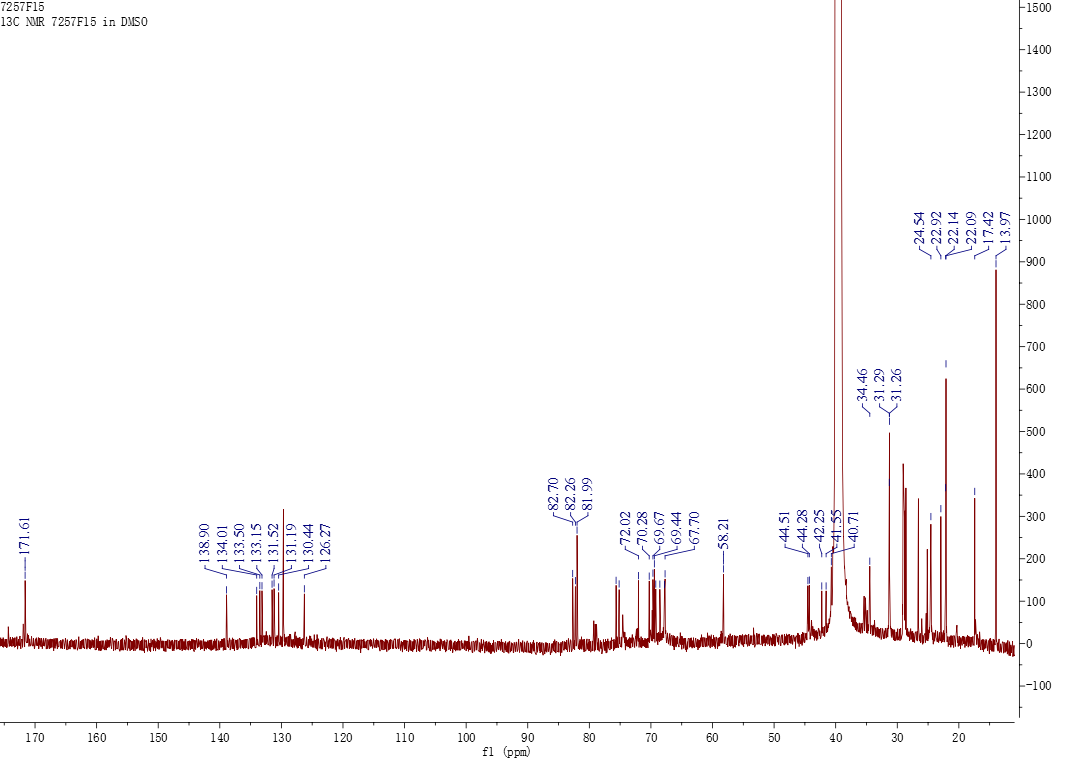
**

**Table S1**

| **Samples** | **Altitude (m)** | **LNG-LAT** | **Color** | **Collection time** | **Soil condition** |
| --- | --- | --- | --- | --- | --- |
| RSP1 | 1962 | 103°26'13.1"E 25°54'33.4"N | Red earth | 9^th^ Nov, 2012 | Healthy PN |
| RSP2 | 1962 | 103°26'13.1"E 25°54'33.4"N | Red earth | 26^th^ Oct, 2013 | Root-rot PN |
| RSP3 | 1980 | 103°31'23.1"E 24°38'1.6"N | Red earth | 9^th^ Nov, 2012 | Root-rot PN |
| RSP4 | 1562 | 23°31'48.9"E 104°19'17.2"N | yellowish red earth | 10^th^ Nov, 2012 | Healthy PN |
| RSP5 | 1562 | 104°19'17.2"E 23°31'48.9"N | yellowish red earth | 13^th^ Dec, 2012 | Serious nematodes infection of PN |
| RSP6 | 1562 | 104°19'17.2"E 23°31'48.9"N | yellowish red earth | 13^th^ Dec, 2012 | Healthy PN |
| RSP7 | 2138 | 103°38'41.3"E 24°44'8.8"N | Red earth | 9^th^ Nov, 2012 | Healthy PN |
| RSP8 | 2138 | 103°38'41.3"E 24°44'8.8"N | Red earth | 9^th^ Nov, 2012 | Root-rot PN |
| RSP9 | 1477 | 103°37'55.0"E 23°48'6.2"N | Red earth | 10^th^ Nov, 2012 | Healthy PN |
| RSP10 | 1494 | 103°37'55.0"E 23°48'6.2"N | Red earth | 8^th^ Nov, 2013 | Healthy PN |
| RSP11 | 647 | 106º 04' 60"E 23º 53' 48"N | Red earth | 26^th^ Feb, 2015 | Healthy PN |
| RSP12 | 1466 | 103°40' E 23°46' N | Red earth | 24^th^ Nov, 2015 | Healthy PN |

**Abbreviations:** PN: *Panax notoginseng*; LNG-LAT: Longitude-latitude; None: No detailed record.

**Table S2**

| **Primer** | **Sequence (5’–3’)** | **Target gene** | **Length of target**  **gene fragment (bp)** | **Protein domain** | **Reference** |
| --- | --- | --- | --- | --- | --- |
| 27F | AGAGTTTGATCCTGGC | 16S rRNA | 1,400~1,500 | 16S ribosomal RNA | Zhang et al. 2014 |
| 1492R | GGTTACCTTGTTACGACTT |
| KSF | GCGATGGATCCNCAGCAGCG | PKSI | ~700 | Ketosynthase (KS) | Zhu et al. 2009 |
| KSR | GTGCCGGTNCCGTGNGYYTC |
| KSIIF | CTGCTTCGACGCCATCAAGG | PKSII | ~600 | Ketosynthase (KS) | Ning-Yu et al. 2015 |
| KSIIR | GAATCCGCCGAAGCCGCT |
| A3F | GCSTACSYSATSTACACSTCSGG | NRPS | 700~800 | Adenylation (A) | Ayuso-Sacido and Genilloud 2005 |
| A7R | SASGTCVCCSGTSCGGTAS |

**Table S3**

| **Isolate** | **Accession number** | **Closest cultivated species (GenBank accession no.)** | **Similarity (%)** | **Samples** |
| --- | --- | --- | --- | --- |
| SYP-A7028 | MH041277 | *Streptomyces microflavus* ATCC 13231^T^ (DQ445795) | 100 | RSP1 |
| SYP-A7030 | MH041278 | *Streptomyces niveus* ATCC 19793 ^T^ (DQ442532) | 99.66 | RSP1 |
| SYP-A7031 | MH041279 | *Streptomyces intermedius* ATCC 3329 ^T^ (AB184277) | 100 | RSP3 |
| SYP-A7038 | MH041280 | *Streptomyces rubiginosohelvolus* ATCC 19926 ^T^ (AB184240) | 100 | RSP4 |
| SYP-A7039 | MH041281 | *Streptomyces rectiviolaceus* ATCC 43690 ^T^ (DQ026660) | 99.85 | RSP4 |
| SYP-A7049 | MH041282 | *Streptomyces fulvissimus* ATCC 27431 ^T^ (AF361784) | 100 | RSP9 |
| SYP-A7053 | MH041283 | *Streptomyces viridosporus* ATCC 27479 ^T^ (DQ442556) | 97.24 | RSP9 |
| SYP-A7055 | MH041284 | *Streptomyces hygroscopicus subsp. glebosus* ATCC 14607 ^T^ (AB184479) | 100 | RSP4 |
| SYP-A7076 | MH041285 | *Streptomyces violaceoruber* ATCC 14980 ^T^ (AF503492) | 100 | RSP1 |
| SYP-A7077 | MH041286 | *Streptomyces coelescens* ATCC 19830 ^T^ (AF503496) | 100 | RSP1 |
| SYP-A7079 | MH041287 | *Streptomyces drozdowiczii* JCM 13580 ^T^ (AB249957) | 100 | RSP7 |
| SYP-A7085 | MH041289 | *Streptomyces bungoensis* DSM 41781 ^T^ (AB184696) | 99.36 | RSP9 |
| SYP-A7087 | MH041290 | *Streptomyces anulatus* ATCC 27416 ^T^ (DQ026637) | 99.83 | RSP5 |
| SYP-A7090 | MH041291 | *Streptomyces purpeofuscus* DSM 40283 ^T^ (AJ781364) | 99.19 | RSP9 |
| SYP-A7096 | MH041292 | *Streptomyces mexicanus* DSM 41796 ^T^ (AB249966) | 98.83 | RSP7 |
| SYP-A7103 | MH041293 | *Streptomyces katrae* ATCC 27440 ^T^ (AB184409) | 99.55 | RSP4 |
| SYP-A7113 | MH041294 | *Streptomyces glauciniger* JCM 12278 ^T^ (AB249964) | 99.39 | RSP7 |
| SYP-A7114 | MH041295 | *Streptomyces misionensis* ATCC 14991 ^T^ (EF178678) | 100 | RSP7 |
| SYP-A7131 | MH045724 | *Streptomyces cyaneofuscatus* ATCC 19746 ^T^ (AB184860) | 100 | RSP9 |
| SYP-A7157 | MH041296 | *Streptomyces hydrogenans* ATCC 19631 ^T^ (AB184868) | 99.39 | RSP5 |
| SYP-A7160 | MH045725 | *Streptomyces badius* ATCC 19729 ^T^ (AY999783) | 100 | RSP4 |
| SYP-A7161 | MH045726 | *Streptomyces glomeroaurantiacus* ATCC 15866 ^T^ (AB249983) | 99.70 | RSP6 |
| SYP-A7178 | MH045727 | *Streptomyces humiferus* ATCC 15719 ^T^ (AF503491) | 100 | RSP8 |
| SYP-A7185 | MH045728 | *Streptomyces longisporoflavus* ATCC 19781 ^T^ (DQ442520) | 99.27 | RSP4 |
| SYP-A7193 | MH041298 | *Streptomyces coelicoflavus* DSM 41471 ^T^ (AB184650) | 100 | RSP1 |
| SYP-A7200 | MH041299 | *Streptomyces hundungensis* JCM 17577 ^T^ (JN560157) | 99.13 | RSP5 |
| SYP-A7201 | MH041300 | *Streptomyces novaecaesareae* ATCC 27452 ^T^ (AB184357) | 99.75 | RSP5 |
| SYP-A7212 | MH041301 | *Streptomyces tricolor* DSM 41704 ^T^ (AB184687) | 100 | RSP7 |
| SYP-A7234 | MH045729 | *Streptomyces albolongus* DSM 40570 ^T^ (AB184425) | 98.99 | RSP9 |
| SYP-A7255 | MH045730 | *Streptomyces globosus* ATCC 14979 ^T^ (AJ781330) | 99.31 | RSP9 |
| SYP-A7257 | MH041302 | *Streptomyces griseofuscus* ATCC 23916 ^T^ (AB184206) | 100 | RSP9 |
| SYP-A7260 | MH045731 | *Streptomyces rubidus* JCM 13277 ^T^ (AY876941) | 99.52 | RSP10 |
| SYP-A7261 | MH041303 | *Streptomyces celluloflavus* ATCC 29806 ^T^ (AB184476) | 99.51 | RSP10 |
| SYP-A7283 | MH041304 | *Streptomyces yatensis* DSM 41771 ^T^ (AB249962) | 99.82 | RSP2 |
| SYP-A7284 | MH155969 | *Streptomyces violaceoruber* ATCC 14980 ^T^ (AF503492) | 100 | RSP2 |
| SYP-A7748 | MH041305 | *Streptomyces panaciradicis* NBRC 109811 ^T^ (KF971876) | 99.06 | RSP11 |
| SYP-A7750 | MH041306 | *Streptomyces luteosporeus* ATCC 33049 ^T^ (DQ442525) | 99.27 | RSP11 |
| SYP-A7752 | MH041307 | *Streptomyces bingchenggensis* BCW-1 ^T^ (CP002047) | 99.66 | RSP11 |
| SYP-A8135 | MH041308 | *Streptomyces caniferus* ATCC 43699 ^T^ (AB184640) | 98.44 | RSP12 |
| SYP-A8136 | MH041309 | *Streptomyces ramulosus* ATCC 19802 ^T^ (DQ026662) | 99.71 | RSP12 |
| SYP-A8194 | MH041310 | *Streptomyces albogriseolus* ATCC 23875 ^T^ (AY177662) | 100 | RSP12 |
| SYP-A8195 | MH041311 | *Streptomyces afghaniensis* ATCC 23871 ^T^ (AB184847) | 100 | RSP12 |

**Table S4**

| **Strain** | **Genbank accession no.** | **BLAST match**  **(accession no.)** | **Representative sequences under BLAST match** | **% identity** | |
| --- | --- | --- | --- | --- | --- |
| **PKS I** | | | | | |
| SYP-A7028 | MH151922 | ACG70841 | polyketide synthase from *Bacillus* sp. WPySW2 | 99 | |
| SYP-A7030 | MH198457 | AWW22989 | polyketide synthase from *Bacillus amyloliquefaciens* | 100 | |
| SYP-A7031 | MH151921 | AWW22983 | polyketide synthase from *Bacillus subtilis* | 99 | |
| SYP-A7038 | MH198458 | PPA44229 | hypothetical protein from *Streptomyces griseus* | 100 | |
| SYP-A7039 | MH198459 | PPA44229 | hypothetical protein from *Streptomyces griseus* | 98 | |
| SYP-A7049 | MH198460 | ACG70841 | polyketide synthase from *Bacillus* sp. WPySW2 | 99 | |
| SYP-A7076 | MH198461 | ACG70841 | polyketide synthase from *Bacillus* sp. WPySW2 | 99 | |
| SYP-A7077 | MH198462 | AGL92436 | polyketide synthase from *Bacillus* sp. SWI6 | 99 | |
| SYP-A7079 | MH198463 | AWW22989 | polyketide synthase from *Bacillus amyloliquefaciens* | 100 | |
| SYP-A7085 | MH198464 | ACG70841 | polyketide synthase from *Bacillus* sp. WPySW2 | 98 | |
| SYP-A7087 | MH198465 | ACI24640 | putative beta-ketoacyl synthase from *Streptomyces* sp. MP9E12 | 95 | |
| SYP-A7103 | MH198466 | AWW22989 | polyketide synthase from *Bacillus amyloliquefaciens* | 99 | |
| SYP-A7131 | MH198467 | ACI24640 | putative beta-ketoacyl synthase from *Streptomyces* sp. MP9E12 | 94 | |
| SYP-A7161 | MH198468 | AWW22983 | polyketide synthase from *Bacillus subtilis* | 99 | |
| SYP-A7178 | MH198469 | AWW22989 | polyketide synthase from *Bacillus amyloliquefaciens* | 99 | |
| SYP-A7185 | MH198470 | AWW22989 | polyketide synthase from *Bacillus amyloliquefaciens* | 99 | |
| **SYP-A7200*** | **MH198471** | **ABJ97439** | **MerC (meridamycin) from *Streptomyces violaceusniger*** | **94** | |
| SYP-A7201 | MH198484 | AWW22989 | polyketide synthase from *Bacillus amyloliquefaciens* | 99 | |
| SYP-A7234 | MH198472 | AWW22989 | polyketide synthase from *Bacillus amyloliquefaciens* | 98 | |
| SYP-A7255 | MH198474 | BAH67004 | Polyketide synthase from *Streptomyces cinnamoneus* subsp. *sparsus* | 89 | |
| **SYP-A7257*** | **MH198473** | **ABJ97439** | **MerC (meridamycin) from *Streptomyces violaceusniger*** | **93** | |
| SYP-A7260 | MH198475 | AWW22989 | polyketide synthase from *Bacillus amyloliquefaciens* | 99 | |
| SYP-A7261 | MH198476 | PJJ00082 | polyketide synthase PksN from *Streptomyces* sp. 2333.5 | 97 | |
| **SYP-A7283*** | **MH198477** | **ABJ97439** | **MerC (meridamycin) from *Streptomyces violaceusniger*** | **94** | |
| SYP-A7284 | MH198478 | ACG70841 | polyketide synthase from *Bacillus* sp. WPySW2 | 98 | |
| SYP-A7750 | MH198479 | AWW22989 | polyketide synthase from *Bacillus amyloliquefaciens* | 100 | |
| SYP-A8135 | MH198480 | AWW22989 | polyketide synthase from *Bacillus amyloliquefaciens* | 100 | |
| SYP-A8136 | MH198481 | ACG70841 | polyketide synthase from *Bacillus* sp. WPySW2 | 98 | |
| SYP-A8194 | MH198482 | AWW22989 | polyketide synthase from *Bacillus amyloliquefaciens* | 99 | |
| **SYP-A8195*** | **MH198483** | **CUI25744** | **Polyketide synthase/Nonribosomal peptide synthase (thiotetronate antibiotics) *Streptomyces thiolactonus*** | **96** | |
| **PKS II** | | | | | |
| SYP-A7028 | MH198485 | ADI24416 | putative ketoacyl synthase from *Streptomyces* sp. HB100 | 83 | |
| SYP-A7030 | MH198525 | BAH67643 | polyketide synthase from *Streptomyces* sp. ID05-A0065 | 80 | |
| SYP-A7031 | MH198526 | BAH67917 | polyketide synthase from *Streptomyces* sp. ID05-A0318 | 82 | |
| SYP-A7038 | MH198486 | ABM91087 | ketoacyl synthase alpha subunit from *Streptomyces lydicus* | 90 | |
| SYP-A7039 | MH198487 | ABM91087 | ketoacyl synthase alpha subunit from *Streptomyces lydicus* | 89 | |
| SYP-A7049 | MH198488 | ABM91087 | ketoacyl synthase alpha subunit from *Streptomyces lydicus* | 89 | |
| SYP-A7053 | MH198527 | ALU57818 | type II polyketide synthase from *Streptomyces* sp. YIM 77524 | 95 |  |
| SYP-A7055 | MH198489 | ADI24416 | putative ketoacyl synthase from *Streptomyces* sp. HB100 | 97 | |
| SYP-A7076 | MH198490 | AFO70121 | Ketosynthase from *Streptomyces carnosus* | 98 | |
| SYP-A7077 | MH198491 | AFO70121 | Ketosynthase from *Streptomyces carnosus* | 98 | |
| SYP-A7085 | MH198492 | ADI24416 | putative ketoacyl synthase from *Streptomyces* sp. HB100 | 93 | |
| SYP-A7087 | MH198493 | BAF43363 | Ketosynthase from *Streptomyces nodosus* subsp. *asukaensis* | 90 | |
| SYP-A7090 | MH198494 | WP_010048567 | beta-ketoacyl-synthase family protein from *Streptomyces chartreusis* | 98 | |
| SYP-A7096 | MH198495 | BAI44069 | ketosynthase, from *Streptomyces s*p. Sp080513GE-23 | 96 | |
| SYP-A7103 | MH198530 | AIE76940 | putative ketosynthase alpha GrhA from *Streptomyces* sp. N48+ | 77 | |
| SYP-A7113 | MH198496 | ABM91087 | ketoacyl synthase alpha subunit from *Streptomyces lydicus* | 90 | |
| SYP-A7114 | MH198497 | WP_111583916 | beta-ketoacyl synthase family protein from *Streptomyces* sp. PsTaAH-130 | 98 | |
| **SYP-A7131*** | **MH198498** | **AGK78904** | **actinorhodin polyketide beta-ketoacyl synthase alpha subunit from *Streptomyces fulvissimus* DSM 40593** | 90 | |
| SYP-A7157 | MH198499 | ABM91087 | ketoacyl synthase alpha subunit from *Streptomyces lydicus* | 88 | |
| SYP-A7160 | MH198500 | AUD55815 | polyketide synthase type-II from *Streptomyces* sp. | 100 | |
| SYP-A7178 | MH198501 | ABM91087 | ketoacyl synthase alpha subunit from *Streptomyces lydicus* | 92 | |
| SYP-A7193 | MH198502 | WP_055422251 | beta-ketoacyl synthase family protein from *Streptomyces pactum* | 97 | |
| SYP-A7200 | MH198503 | WP_114035345 | beta-ketoacyl synthase family protein from *Streptomyces* sp. SDr-06 | 95 | |
| SYP-A7201 | MH198504 | WP_030743825 | beta-ketoacyl synthase family protein from *Streptomyces* sp. NRRL S-31 | 90 | |
| SYP-A7212 | MH198505 | AFO70121 | Ketosynthase from *Streptomyces carnosus* | 98 | |
| SYP-A7234 | MH198506 | WP_010048567 | beta-ketoacyl-synthase family protein from *Streptomyces chartreusis* | 97 | |
| SYP-A7255 | MH198507 | ABM91087 | ketoacyl synthase alpha subunit from *Streptomyces lydicus* | 93 | |
| SYP-A7257 | MH198508 | BAF43341 | Ketosyntahse from *Streptomyces roseocinereus* | 85 | |
| SYP-A7260 | MH198509 | ABM91087 | ketoacyl synthase alpha subunit from *Streptomyces lydicus* | 93 | |
| SYP-A7261 | MH198510 | AFO70121 | Ketosynthase from *Streptomyces carnosus* | 89 | |
| SYP-A7283 | MH198511 | ABM91087 | ketoacyl synthase alpha subunit from *Streptomyces lydicus* | 88 | |
| SYP-A7284 | MH198512 | AFO70121 | Ketosynthase from *Streptomyces carnosus* | 89 | |
| SYP-A7748 | MH198513 | ABM91087 | ketoacyl synthase alpha subunit from *Streptomyces lydicus* | 94 | |
| **SYP-A7750*** | **MH198514** | **AGK78904** | **actinorhodin polyketide beta-ketoacyl synthase alpha subunit from *Streptomyces fulvissimus* DSM 40593** | **90** | |
| SYP-A8135 | MH198515 | APD71706 | type II polyketide synthase from *Streptomyces* sp. MM24 | 90 | |
| SYP-A8136 | MH198516 | APD71706 | type II polyketide synthase from *Streptomyces* sp. MM24 | 90 | |
| SYP-A8194 | MH198517 | APD71706 | type II polyketide synthase from *Streptomyces* sp. MM24 | 90 | |
| SYP-A8195 | MH198518 | WP_020275095 | beta-ketoacyl synthase family protein from *Streptomyces afghaniensis* | 98 |  |
| **NRPS** | | | | | |
| SYP-A7055 | MH198519 | WP_109886510 | non-ribosomal peptide synthetase from *Streptomyces* sp. NEAU-S7GS2 | 97 | |
| SYP-A7076 | MH198520 | EFD71468 | non-ribosomal peptide synthetase from *Streptomyces lividans* TK24 | 99 | |
| SYP-A7087 | MH198528 | WP_109878283 | non-ribosomal peptide synthetase from *Streptomyces* sp. FT05W | 95 | |
| SYP-A7157 | MH198529 | WP_030696838 | hybrid non-ribosomal peptide synthetase/type I polyketide synthase from *Streptomyces griseus* | 93 | |
| SYP-A7193 | MH198521 | WP_087788854 | non-ribosomal peptide synthetase from *Streptomyces* sp. CS159 | 94 | |
| SYP-A7255 | MH198522 | KOU34160 | peptide synthetase from *Streptomyces* sp. WM6378 | 92 | |
| SYP-A7750 | MH198523 | AWS21996 | non-ribosomal peptide synthetase from *Streptomyces* sp. | 93 | |

**Note: Strains marked with * were selected for small-scale fermentation and HPLC-DAD analysis.**

**Table S5**

|  | **a-ring** | |  | **b-ring** | |
| --- | --- | --- | --- | --- | --- |
| **assignment** | **Position** | ***δ* C, type** | **assignment** | **Position** | ***δ* C, type** |
| **Thr** | **1** | 168.8, C | **Thr** | **1** | 169.0, C |
|  | **2** | 54.9, CH |  | **2** | 55.1, CH |
|  | **3** | 74.9, CH |  | **3** | 74.8, CH |
|  | **4** | 17.4 CH_3_ |  | **4** | 17.8, CH_3_ |
| **Val** | **1** | 173.7, C | **Val** | **1** | 174.2, C |
|  | **2** | 58.7, CH |  | **2** | 57.3, CH |
|  | **3** | 31.8, CH |  | **3** | 32.0, CH |
|  | **3-Me** | 19.2, 19.4, CH_3_ |  | **3-Me** | 19.2, 19.3, CH_3_ |
| **R-Pro** | **1** | 172.8, C | **R-Pro** | **1** | 173.3, C |
|  | **2** | 56.5, CH |  | **2** | 54.4, CH |
|  | **3** | 31.1, CH_2_ |  | **3** | 42.0, CH_2_ |
|  | **4** | 22.8, CH_2_ |  | **4** | 209.0, C |
|  | **5** | 47.5, CH_2_ |  | **5** | 53.0, CH_2_ |
| **Sar** | **1** | 167.9, C | **Sar** | **1** | 167.7, C |
|  | **2** | 51.4, CH_2_ |  | **2** | 51.4, CH_2_ |
|  | **N-Me** | 34.9, CH_3_ |  | **N-Me** | 35.0, CH_3_ |
| **Me-Val** | **1** | 166.4, C | **Me-Val** | **1** | 166.6, C |
|  | **2** | 71.4, CH |  | **2** | 71.6, CH |
|  | **3** | 29.8, CH |  | **3** | 29.8, CH |
|  | **3-Me** | 18.9, 21.7, CH_3_ |  | **3-Me** | 19.1, 21.8, CH_3_ |
|  | **N-Me** | 39.3, CH_3_ |  | **N-Me** | 39.5, CH_3_ |
| **Chromophore** | **1** | 101. 9, C |  |  |  |
|  | **2** | 147.5, C |  |  |  |
|  | **3** | 179.7, C |  |  |  |
|  | **4** | 113.7, C |  |  |  |
|  | **4a** | 145.1, C |  |  |  |
|  | **5a** | 140.6, C |  |  |  |
|  | **6** | 128.0, C |  |  |  |
|  | **7** | 130.4, CH |  |  |  |
|  | **8** | 126.3, CH |  |  |  |
|  | **9** | 132.2, C |  |  |  |
|  | **9a** | 129.3, C |  |  |  |
|  | **10a** | 146.0, C |  |  |  |
|  | **4-Me** | 7.9, CH_3_ |  |  |  |
|  | **6-Me** | 14.2, CH_3_ |  |  |  |
|  | **1-CO** | 166.1, C |  |  |  |
|  | **9-CO** | 166.0, C |  |  |  |

**Table S6**

| **Position** | ***δ* C, type** | ***δ* H (*J* in Hz)** | **Position** | ***δ* C, type** | ***δ* H (*J* in Hz)** |
| --- | --- | --- | --- | --- | --- |
| **1** | 173.0, C | - | **19** | 135.4, CH | 6.28~6.51, 1H, m |
| **2** | 60.5, CH | 2,56, 1H, dd, *J*=6.0 | **20** | 134.1, CH | 6.28~6.51, 1H, m |
| **3** | 73.3, CH | 4.03, 1H, m | **21** | 134.8, CH | 6.28~6.51, 1H, m |
| **4** | 41.1, CH_2_ | 1.33~1.61, 2H, m | **22** | 133.6, CH | 6.28~6.51, 1H, m |
| **5** | 74.2, CH | 4.02, 1H, m | **23** | 134.1, CH | 6.28~6.51, 1H, m |
| **6** | 45.2, CH_2_ | 1.33~1.61, 2H, m | **24** | 131.9, CH | 6.28~6.51, 1H, m |
| **7** | 74.0, CH | 4.18, 1H, m | **25** | 134.3, CH | 6.06, 1H, dd, *J*=12.0, 6.0 |
| **8** | 45.3, CH_2_ | 1.33~1.61, 2H, m | **26** | 73.1, CH | 4.10, 1H, dd, *J*=6.0 |
| **9** | 74.3, CH | 4.02, 1H, m | **27** | 75.2, CH | 4.84, 1H, m |
| **10** | 44.3, CH_2_ | 1.33~1.61, 2H, m | **28** | 17.9, CH_3_ | 1.29, 2H, d, *J*=6.0 |
| **11** | 71.5, CH | 3.97, 1H, m | **29** | 11.7, CH_3_ | 1.78, 3H, s |
| **12** | 39.5, CH_2_ | 1.33~1.61, 2H, m | **1’** | 72.4, CH | 3.84, 1H, m |
| **13** | 70.2, CH | 3.26, 1H, d, *J*=12.0 | **2’** | 36.2, CH_2_ | 1.33~1.61, 2H, m |
| **14** | 78.3, CH | 3.72, 1H, dd, *J*=12.0, 6.0 | **3’** | 26.1, CH_2_ | 1.33~1.61, 2H, m |
| **15** | 80.5, CH | 3.89, 1H, d, *J*=12.0 | **4’** | 33.0, CH_2_ | 1.33~1.61, 2H, m |
| **16** | 138.5, C | - | **5’** | 23.7, CH_2_ | 1.33~1.61, 2H, m |
| **17** | 129.9, CH | 6.02, 1H, dd, *J*=12.0, 6.0 | **6’** | 14.4, CH_3_ | 0.91, t, 3H, *J*=6.0 |
| **18** | 129.0, CH | 6.28~6.51, 1H, m |  |  |  |

**Table S7**

| **Position** | **Compound F7** | | **Compound F8** | |
| --- | --- | --- | --- | --- |
|  | ***δ* C, type** | ***δ* H (*J* in Hz)** | ***δ* C, type** | ***δ* H (*J* in Hz)** |
| **1** | 173.2, C | - | 171.6, C | - |
| **2** | 52.5, CH | 2.25, 1H, m | 58.2, CH | 2.45, 1H, t, 6.0 |
| **3** | 70.8, CH | 3.62, 1H, m | 69.4, CH | 4.02, 1H, m |
| **4** | 41.6, CH_2_ | 1.33, 2H, m | 40.6, CH_2_ | 1.53, 2H, m |
| **5** | 69.5, CH | 3.89, 1H, m | 70.2, CH | 4.70, 1H, m |
| **6** | 43.5, CH_2_ | 1.34, 2H, m | 44.2, CH_2_ | 1.48, 2H, m |
| **7** | 69.6, CH | 3.89, 1H, m | 68.5, CH | 3.96, 1H, m |
| **8** | 43.8, CH_2_ | 1.30, 1.41, 2H, m | 44.4, CH_2_ | 1.51, 2H, m |
| **9** | 71.1, CH | 3.87, 1H, m | 67.7, CH | 3.89, 1H, m |
| **10** | 42.6, CH_2_ | 1.30, 1.38, 2H, m | 42.2, CH_2_ | 1.70, 2H, m |
| **11** | 69.3, CH | 3.80, 1H, m | 67.6, CH | 3.84, 1H, m |
| **12** | 38.7, CH_2_ | 1.59, 1.37, 2H, m | 41.5, CH | 1.60, 2H, m |
| **13** | 68.2, CH | 3.13, 1H, m | 75.1, CH | 3.29, 1H, m |
| **14** | 76.5, CH | 3.49, 1H, m | 82.2, CH | 3.34, 1H, m |
| **15** | 78.0, CH | 3.70, 1H, m | 82.6, CH | 3.64, 1H, m |
| **16** | 138.9, C | - | 81.9, C | - |
| **17** | 127.1, CH | 5.94, 1H, dd, 12.0, 6.0 | 138.8, CH | 5.72, 1H, d, 12.0 |
| **18** | 128.0, CH | 6.47, 1H, dd, 12.0, 6.0 | 126.2, CH | 6.19, 1H, dd, 12.0, 6.0 |
| **19** | 133.0, CH | 6.27, 1H, m | 133.9, CH | 5.66, 1H, m |
| **20** | 132.5, CH | 6.35, 1H, m | 131.1, CH | 6.29, 1H, m |
| **21** | 133.1, CH | 6.35, 1H, m | 133.1, CH | 6.26, 1H, m |
| **22** | 131.7, CH | 6.26, 1H, m | 130.4, CH | 6.28, 1H, m |
| **23** | 132.9, CH | 6.33, 1H, m | 131.4, CH | 6.25, 1H, m |
| **24** | 129.1, CH | 6.33, 1H, m | 133.4, CH | 6.25, 1H, m |
| **25** | 135.1, CH | 5.97, 1H, m | 71.9, CH | 4.23, 1H, br, s |
| **26** | 71.3, CH | 3.96, 1H, m | 75.6, CH | 3.50, 1H, m |
| **27** | 73.0, CH | 4.64m 1H, dq, 6.0 | 69.1, CH | 3.96, 1H, m, |
| **28** | 18.1, CH_3_ | 1.22, 3H, d, 6.0 | 17.4, CH_3_ | 1.13, 3H, d, 6.0 |
| **29** | 11.8, CH_3_ | 1.69, 3H, s | 22.8, CH_3_ | 1.06, 3H, s |
| **1’** | 28.6, CH_2_ | 1.66, 1.45, 2H, m | 69.6, CH | 3.67, 1H, m |
| **2’** | 28.8, CH_2_ | 1.28, 1.15, 2H, m | 34.4, CH_2_ | 1.27, 2H, m |
| **3’** | 21.9, CH_2_ | 1.26, 2H, m | 24.5, CH_2_ | 1.23, 2H, m |
| **4’** | 13.9, CH_3_ | 0.85, 3H, t, 6.0 | 31.2, CH_2_ | 1.22, 2H, m |
| **5’** |  |  | 22.1, CH_2_ | 1.23, 2H, m |
| **6’** |  |  | 13.9, CH_3_ | 0.83, 3H, t, 6.0 |

**References:**

Ayuso-Sacido, A. and Genilloud, O. (2005) New PCR Primers for the Screening of NRPS and PKS-I Systems in Actinomycetes: Detection and Distribution of These Biosynthetic Gene Sequences in Major Taxonomic Groups. *MICROB ECOL* **49**, 10-24.

Ning-Yu, J., Fei, P., Fang, Z.K., Wu, Y.D., Chen, M.H., Yang, X., Wang, H.R., Hong, J. and Lian, Y.Y. (2015) Study on aromatic polyketide metabolite with antibacterial activity from the marine-derived Actinomadura sp. FIM95-F26. *Chinese Journal of Antibiotics* **40**, 161-165.

Zhang, M.Y., Xie, J., Zhang, T.Y., Xu, H., Cheng, J., Li, S.H., Li, W.J. and Zhang, Y.X. (2014) Zhang, M. Y. et al. Sinomonas notoginsengisoli sp. nov., isolated from the rhizosphere of Panax notoginseng. Antonie van Leeuwenhoek 106, 827-835. *Antonie Van Leeuwenhoek* **106**.

Zhu, P., Zheng, Y., You, Y., Yan, X. and Shao, J. (2009) Molecular phylogeny and modular structure of hybrid NRPS/PKS gene fragment of Pseudoalteromonas sp. NJ6-3-2 isolated from marine sponge Hymeniacidon perleve. *Journal of Microbiology & Biotechnology* **19**, 229.
